# Somatostatin-type and allatostatin-C–type neuropeptides are paralogous and have opposing myoregulatory roles in an echinoderm

**DOI:** 10.1073/pnas.2113589119

**Published:** 2022-02-10

**Authors:** Ya Zhang, Luis Alfonso Yañez-Guerra, Ana B. Tinoco, Nayeli Escudero Castelán, Michaela Egertová, Maurice R. Elphick

**Affiliations:** ^a^School of Biological and Behavioural Sciences, Queen Mary University of London, London, E1 4NS, United Kingdom

**Keywords:** neuropeptide, evolution, somatostatin, allatostatin-C, starfish

## Abstract

Somatostatin (SS) and allatostatin-C (ASTC) are inhibitory neuropeptides in chordates and protostomes, respectively, which hitherto were identified as orthologs. However, echinoderms have two SS/ASTC-type neuropeptides (SS1 and SS2), and here, our analysis of sequence data indicates that SS1 is an ortholog of ASTC and SS2 is an ortholog of SS. The occurrence of both SS-type and ASTC-type neuropeptides in echinoderms provides a unique context to compare their physiological roles. Investigation of the expression and actions of the ASTC-type neuropeptide ArSS1 in the starfish *Asterias rubens* revealed that it causes muscle contraction (myoexcitation), contrasting with myoinhibitory effects of the SS-type neuropeptide ArSS2. Our findings suggest that SS-type and ASTC-type neuropeptides are paralogous and originated by gene duplication in a common ancestor of the Bilateria, with only one type being retained in chordates (SS) and protostomes (ASTC) but with both types being retained in echinoderms. Loss of ASTC-type and SS-type neuropeptides in chordates and protostomes, respectively, may have been due to their functional redundancy as inhibitory regulators of physiological processes. Conversely, the retention of both neuropeptide types in echinoderms may be a consequence of the evolution of a myoexcitatory role for ASTC-type neuropeptides mediated by as yet unknown signaling mechanisms.

## Significance

Somatostatin (SS) and allatostatin-C (ASTC) are related neuropeptide hormones that act as inhibitory regulators of physiological processes in chordates (e.g., humans) and protostome invertebrates (e.g., insects), respectively. We have discovered that echinoderms (e.g., starfish) uniquely have both SS-type and ASTC-type neuropeptides, which act as inhibitory and excitatory regulators of muscle activity, respectively. Our findings suggest that SS-type and ASTC-type neuropeptides evolved by duplication of a common ancestral encoding gene. Then, one of the neuropeptides was lost in protostomes and chordates, probably because of their functional redundancy as inhibitory regulators. Conversely, the unique retention of both neuropeptide types in echinoderms may be explained by evolution of an excitatory role for ASTC-type neuropeptides mediated by yet-to-be-determined signaling mechanisms.

Neuropeptides are neuronally secreted peptides that regulate diverse physiological and behavioral processes in animals, with their effects on target cells/tissues typically mediated by G protein–coupled receptors (1). Investigation of the phylogenetic distribution of neuropeptides and their cognate receptors has revealed that the evolutionary origin of over 30 neuropeptide signaling systems can be traced back to the urbilaterian common ancestor of protostomes and deuterostomes (2–6). Important insights into neuropeptide evolution have been obtained from nonchordate deuterostome invertebrates (e.g., echinoderms), which occupy an “intermediate” phylogenetic position with respect to chordates and protostomes (4, 7, 8). For example, molecular and functional characterization of neuropeptide signaling systems in echinoderms has identified “missing links” that facilitated reconstruction of the evolutionary history of the neuropeptide-S/crustacean cardioactive peptide-type, corazonin-type, and prolactin-releasing peptide/short Neuropeptide-F–type neuropeptide signaling systems in the Bilateria (9–11).

Here, we have investigated the evolution and comparative physiology of somatostatin (SS)-type and allatostatin-C (ASTC)-type peptides—structurally and evolutionarily related neuropeptides in chordates and protostomes, respectively (2, 3). Thus, SS-type and ASTC-type neuropeptides have the common characteristics of containing two cysteine residues that form a disulphide bridge and being derived from the carboxyl-terminal region of their precursor proteins (12–16). Furthermore, the G protein–coupled receptors that mediate the physiological effects of SS-type and ASTC-type neuropeptides in chordates and protostomes, respectively, share sequence similarity indicative of homology (2, 3, 17, 18). Accordingly, it has been proposed that ASTC-type signaling in protostomes is orthologous to SS-type signaling in chordates (19). Interestingly, analysis of transcriptome/genome sequence data from echinoderms has revealed the occurrence of two SS/ASTC-type neuropeptides in species belonging to this phylum, which we have referred to as SS1 and SS2 (8, 20, 21). Furthermore, comparison of the sequences of these peptides with SS/ASTC-type neuropeptides in other bilaterian taxa has revealed that echinoderm SS1-type and SS2-type neuropeptides have specific structural similarities with ASTC-type neuropeptides in some protostomes and SS-type neuropeptides in chordates, respectively (21). This is intriguing because it suggests that echinoderm SS1-type neuropeptides are orthologs of protostome ASTC-type neuropeptides and that echinoderm SS2-type neuropeptides are orthologs of chordate SS-type neuropeptides. To further investigate these relationships, here we present a more extensive comparative analysis of the sequences of SS/ASTC-type neuropeptides in echinoderms and other taxa than previously reported (21). Furthermore, we report use of the cluster analysis of sequences (CLANS) method (22) to compare the sequences of the precursors of SS/ASTC-type neuropeptides in echinoderms and other taxa. In addition, we have employed analysis of gene synteny at a chromosomal level to investigate relationships of SS/ASTC-type neuropeptide precursors in echinoderms and other taxa.

Molecular characterization of SS/ASTC-type neuropeptide signaling in the starfish *Asterias rubens* has determined the mature structures of the SS1-type peptide ArSS1 (KCIGRFQPFSMPC) and the SS2-type peptide ArSS2 (RAKNARCMADFWKGRGLVDC), with both peptides having a disulphide bridge between the underlined cysteine residues, consistent with the structures of SS/ASTC-type peptides in other taxa (21). Furthermore, ArSS2, but not ArSS1, acts as a ligand for three G protein–coupled receptors (ArSSR1-3) that are co-orthologs of SS-type receptors in chordates (21). Detailed analysis of the anatomical expression pattern and pharmacological actions of ArSS2 in *A. rubens* revealed that it acts as a muscle relaxant and triggers stomach eversion in this species (21). This myoinhibitory effect of ArSS2 in starfish is noteworthy because it is consistent with the effects of SS/ASTC-type neuropeptides as inhibitory regulators of a variety of physiological processes in chordates and protostomes (13, 14, 23–26). Thus, an ancient role of SS/ASTC-type neuropeptides as inhibitory regulators can be traced back to the common ancestor of the Bilateria, with this role being retained in the action of the SS2-type neuropeptide ArSS2 as a myoinhibitor in an echinoderm—the starfish *A. rubens* (21). In this context, it was of interest to determine the physiological roles of SS1-type neuropeptides in echinoderms, and to address this issue here, we have investigated the expression and actions of ArSS1 in the starfish *A. rubens*.

## Results

### Sequence Analysis Indicates That Echinoderm SS1-Type Peptides Are Orthologs of Protostome ASTC-Type Peptides and That Echinoderm SS2-Type Peptides Are Orthologs of Chordate SS-Type Peptides.

**Fig. 1.**
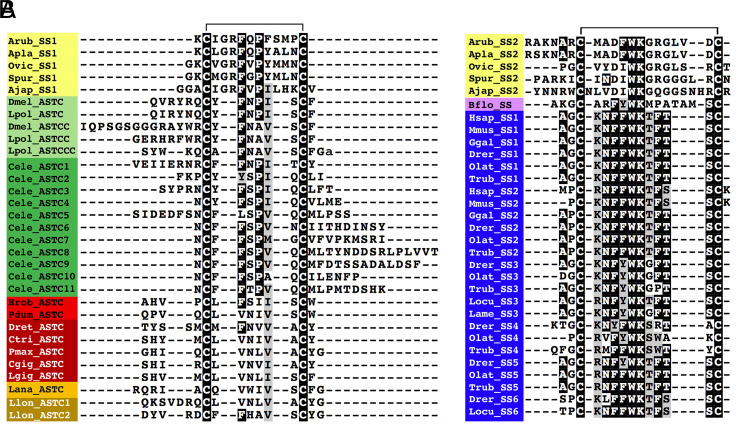
Sequence alignment of echinoderm SS1-type peptides with protostome ASTC-related peptides and echinoderm SS2-type peptides with chordate SS-type peptides. (*A*) Alignment of echinoderm SS1-type neuropeptides with protostome ASTC-type peptides. Note that in addition to conserved cysteine residues, there is a conserved FXP motif (in which X is variable) shared by some echinoderm SS1-type peptides, arthropod ASTC-type peptides, and several *C. elegans* ASTC-type peptides. (*B*) Alignment of echinoderm SS2-type neuropeptides with chordate SS-type peptides. Note that in addition to conserved cysteine residues, there is a conserved Phe-Trp-Lys/Ile-Trp-Lys (FWK/IWK) motif shared by echinoderm SS2-type peptides and chordate SS-type peptides. The aligned amino acids are highlighted in black if the residue is present in at least 60% of the sequences or highlighted in gray if conservative amino acid substitutions are present in at least 60% of the sequences. The position of a disulphide bridge between the two conserved cysteine residues is shown above the alignments. Species and peptide names are highlighted in taxon-specific colors: yellow (Echinodermata), light green (Arthropoda), dark green (Nematoda), light red (Annelida), dark red (Mollusca), light orange (Brachiopoda), dark orange (Nemertea), pink (Cephalochordata), and blue (Vertebrata). Species name abbreviations are as follows: Ajap (*Apostichopus japonicus*), Apla (*A. planci*), Arub (*A. rubens*), Bflo (*B. floridae*), Cele (*C. elegans*), Cgig (*Crassostrea gigas*), Ctri (*Charonia tritonis*), Dmel (*D. melanogaster*), Drer (*Danio rerio*), Dret (*Deroceras reticulatum*), Ggal (*Gallus gallus*), Hsap (*Homo sapiens*), Hrob (*Helobdella robusta*), Lame (*Lophius americanus*), Lana (*Lingula anatine*), Lgig (*Lottia gigantea*), Llon, (*Lineus longissimus*), Locu (*Lepisosteus oculatus*), Lpol (*Limulus polyphemus*), Mmus (*Mus musculus*), Olat (*Oryzias latipes*), Ovic (*Ophionotus victoriae*), Pdum (*Platynereis dumerilii*), Pmax (*P. maximus*), Spur (*Strongylocentrotus purpuratus*), and Trub (*Takifugu rubripes*). The accession numbers or references for sequences of the neuropeptides included in this figure are listed in *SI Appendix*, Table S1.

Sequence alignments revealed that echinoderm SS1-type and SS2-type peptides have sequence motifs in common with protostome ASTC-type peptides and chordate SS-type peptides, respectively (Fig. 1). Echinoderm SS1-type peptides have a Phe-X-Pro (FXP) motif (in which X is variable) in common with arthropod ASTC-type peptides and several *Caenorhabditis elegans* ASTC-type peptides. However, in arthropod ASTCC/ASTCCC-type peptides and lophotrochozoan ASTC-type peptides, the proline (P) residue is substituted with alanine (A) or valine (V)/leucine (L)/isoleucine (I), respectively. Furthermore, in the sea cucumber *Apostichopus japonicus*, the P is followed by an I residue, which is a characteristic of many protostome ASTC-type peptides (Fig. 1*A*). SS2-type peptides in the starfish *A. rubens* and *Acanthaster planci* have a Phe-Trp-Lys (FWK) motif, which is a conserved characteristic in many SS-type peptides in chordates, whereas in other echinoderm species analyzed, the phenylalanine (F) is substituted with an I residue. Furthermore, in all of the echinoderm SS2-type peptides analyzed, the lysine (K) residue is followed by a glycine residue, which is also a characteristic of some SS-type peptides in teleost fish (Fig. 1*B*). Collectively, these findings provide further evidence that echinoderm SS1-type peptides are orthologs of protostome ASTC-type peptides and that echinoderm SS2-type peptides are orthologs of chordate SS-type peptides.

**Fig. 2.**
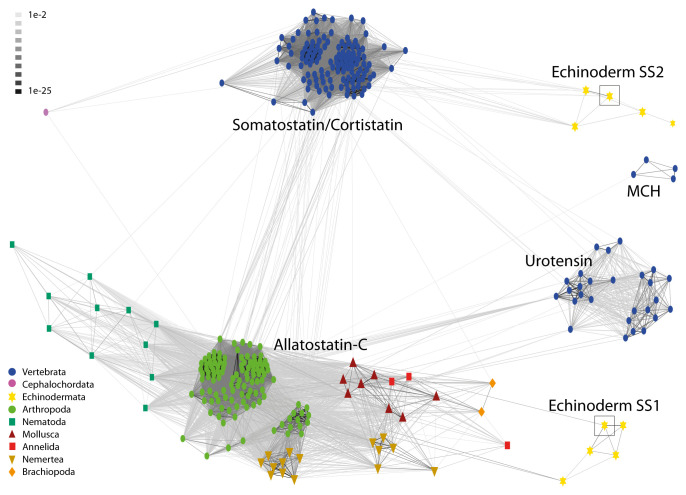
BLOSUM62 cluster map showing sequence similarity of echinoderm SS1-type and SS2-type peptide precursors with SS/ASTC-type peptide precursors in other taxa. Nodes are labeled with phylum-specific colors, as shown in the key, and connections represent BLAST relationships with a *P* value >1e-2. Vertebrate precursors of UII, URP-type peptides, and MCH, which are structurally and evolutionarily related to SS/ASTC-type neuropeptides, were also included for comparison. Note that echinoderm SS1-type peptide precursors only have connections with protostome ASTC-type precursors, whereas echinoderm SS2-type peptide precursors only have connections with vertebrate SS/CST-type precursors. This indicates that echinoderm SS1-type peptide precursors are orthologs of protostome ASTC-type peptide precursors and that echinoderm SS2-type peptide precursors are orthologs of vertebrate SS/CST-type precursors. The *A. rubens* SS1 precursor (ArSSP1) and SS2 precursor (ArSSP2) are boxed. The accession numbers or references for sequences of the precursors included in this figure are listed in *SI Appendix*, Table S2.

To investigate relationships of echinoderm SS1-type and SS2-type peptides with SS/ASTC-type peptides in other taxa, we also analyzed the sequences of the precursor proteins for these peptides using the CLANS method, including precursors of vertebrate SS-related peptides (urotensin II, UII; urotensin-related peptide, URP; and melanin-concentrating hormone, MCH) as out-groups. This revealed that vertebrate SS-type, vertebrate UII/URP-type, vertebrate MCH-type, and protostome ASTC-type precursors form four distinct clusters. Furthermore, and notably, echinoderm SS1-type precursors only showed links with protostome ASTC-type precursors, and echinoderm SS2-type precursors only showed links with vertebrate SS-type precursors (Fig. 2). Consistent with evidence from neuropeptide sequence alignments (Fig. 1), these findings suggest that echinoderm SS1-type neuropeptides are orthologs of protostome ASTC-type neuropeptides and that echinoderm SS2-type neuropeptides are orthologs of vertebrate SS-type neuropeptides.

**Fig. 3.**
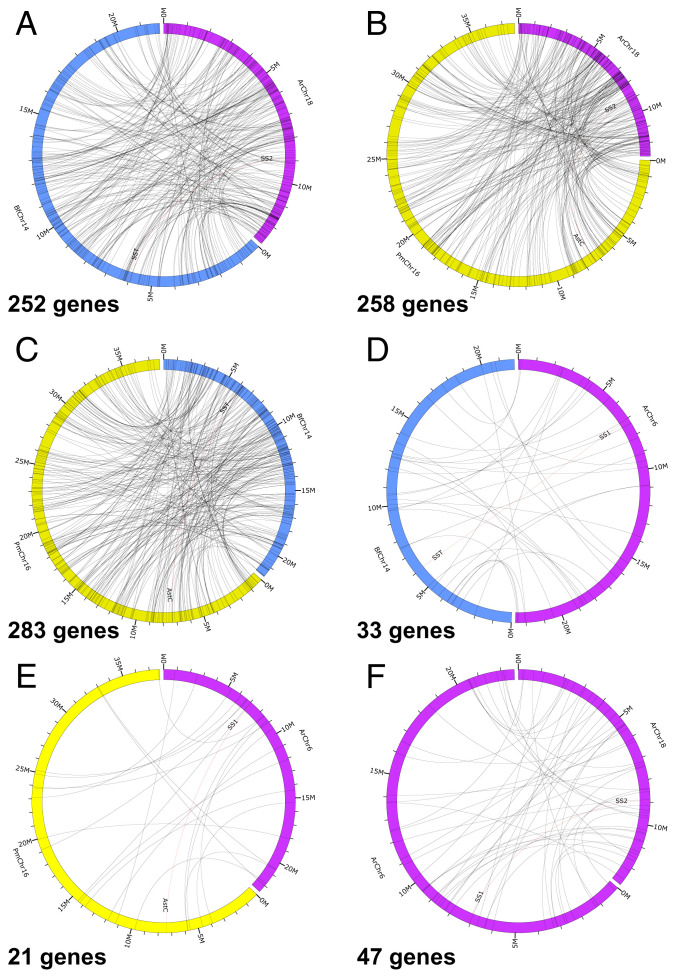
Comparison of the number of putative orthologous genes on chromosomes containing the ArSSP1 and ArSSP2 genes in *A. rubens* and chromosomes containing the SS-type precursor gene in the cephalochordate *B. floridae* and the ASTC-type precursor gene in the mollusk *P. maximus.* (*A*) ArChr18 (contains SS2 gene) compared with *B. floridae* chromosome 14 (BfChr14; contains SST gene) = 252 putative orthologs. (*B*) *A. rubens* chromosome 18 (ArChr18; contains SS2 gene) compared with *P. maximus* chromosome 16 (PmChr16; contains ASTC gene) = 258 putative orthologs. (*C*) *B. floridae* chromosome 14 (BfChr14; contains SST gene) compared with *P. maximus* chromosome 16 (PmChr16; contains ASTC gene) = 283 putative orthologs. (*D*) *A. rubens* chromosome 6 (ArChr6; contains SS1 gene) compared with *B. floridae* chromosome 14 (BfChr14; contains SST gene) = 33 putative orthologs. (*E*) *A. rubens* chromosome 6 (ArChr6; contains SS1 gene) compared with *P. maximus* chromosome 16 (PmChr16; contains ASTC gene) = 21 putative orthologs. (*F*). *A. rubens* chromosome 18 (ArChr18; contains SS2 gene) compared with *A. rubens* chromosome 6 (ArChr6; contains SS1 gene) = 44 putative orthologs. This intraspecies chromosomal analysis provides a comparator for the interspecies chromosomal analysis in *A*–*E*. Chromosome lengths and gene positions can be inferred from markers at 1- and 5-Megabase (M) intervals. The large numbers of putative orthologs identified in the comparisons in *A* (252), *B* (258), and *C* (283) provide evidence that the *A. rubens* SS2 precursor gene, *B. floridae* SS precursor gene, and *P. maximus* ASTC precursor gene are located on chromosomes that are extensively derived from the urbilaterian chromosome that contained a gene that is the common ancestor of bilaterian SS/ASTC-type precursor genes. Conversely, the smaller number of putative orthologs in *D* (33) and *E* (21) indicates that the ArSSP1 gene is located on a chromosome that is to a much lesser extent derived from the urbilaterian chromosome that contained a gene that is the common ancestor of bilaterian SS/ASTC-type precursor genes.

To further investigate orthology, we analyzed the gene repertoires of the chromosomes containing the genes encoding ArSSP1 (chromosome 6; ArChr6) and ArSSP2 (chromosome 18; ArChr18) in *A. rubens* and compared with the gene repertoires of chromosomes containing the SS-type precursor gene in the chordate *Branchiostoma floridae* (chromosome 14; BfChr14) and the ASTC-type precursor gene in the mollusk *Pecten maximus* (chromosome 16; PmChr16). *B. floridae* and *P. maximus* were selected because both species have been shown to have a high level of conservation of the predicted urbilaterian karyotype (27–29). Our analysis revealed that ArChr18 (chromosome with ArSSP2 gene) contains 252 genes and 258 genes that are putative orthologs (based on reciprocal basic local alignment search tool [BLAST] analysis) of genes located on BfChr14 and PmChr16, respectively (Fig. 3 *A* and *B* and Dataset S1). Likewise, BfChr14 and PmChr16 contain 283 genes that are putative orthologs (based on reciprocal BLAST) (Fig. 3*C*). In contrast, ArChr6 (chromosome with ArSSP1 gene) contains only 33 genes and 21 genes that are putative orthologs (based on reciprocal BLAST) of genes located on BfChr14 and PmChr16, respectively (Fig. 3 *D* and *E*). These findings indicate that the *A. rubens* SS2 precursor gene, *B. floridae* SS precursor gene, and *P. maximus* ASTC precursor gene are located on chromosomes that are extensively derived from a common ancestral urbilaterian chromosome, which we can infer would have contained a gene that is the common ancestor of bilaterian SS/ASTC-type precursor genes. Conversely, the relatively smaller number genes on the ArSSP1 gene–containing chromosome in *A. rubens* (ArChr6) that have orthologs on BfChr14 and PmChr16 indicate that the ArSSP1 gene is located on a chromosome that is to a far lesser extent derived from the urbilaterian chromosome that contained the common ancestor of bilaterian SS/ASTC-type precursor genes.

### Localization of ASTC-Type Precursor (ArSSP1) and Peptide (ArSS1) Expression in *A. rubens*.

Informed by evidence that echinoderms have both ASTC-type (SS1) and SS-type (SS2) neuropeptides, we then investigated the expression and pharmacological effects of ArSS1 in *A. rubens* to enable comparison with previously reported findings for ArSS2 (21).

**Fig. 4.**
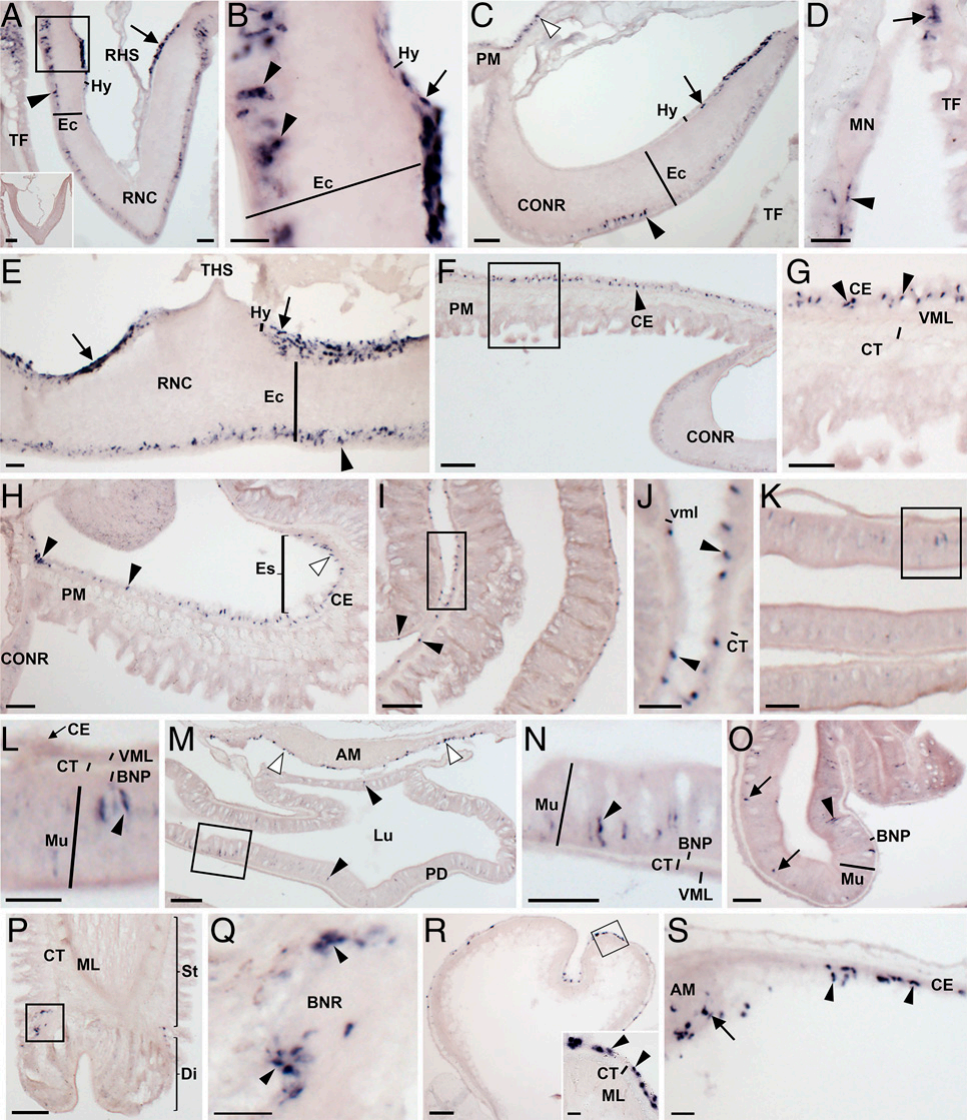
Localization of ArSSP1 mRNA in *A. rubens* using in situ hybridization. (*A*) Transverse section of radial nerve cord incubated with antisense probes showing ArSSP1-expressing cells in both the hyponeural region (arrow) and the ectoneural region (arrowhead). Stained cells can also be seen in an adjacent tube foot. The *Inset* shows that no staining is observed in a section incubated with sense probes, demonstrating the specificity of staining observed in sections incubated with antisense probes. (*B*) Higher-magnification image of the boxed region in *A*, showing stained cells in the hyponeural region (arrow) and in the subcuticular epithelial layer of the ectoneural region (arrowheads). (*C*) Transverse section of circumoral nerve ring showing stained cells in the hyponeural region (arrow) and the ectoneural region (arrowhead). Stained cells can also be observed in the coelomic epithelial lining of the peristomial membrane (white arrowhead). (*D*) Stained cells can be seen in the external epithelial layer of the marginal nerve (arrowhead) and in an adjacent tube foot (arrow). (*E*) Parasagittal longitudinal section of radial nerve cord showing stained cells in both the hyponeural region (arrows) and ectoneural region (arrowhead). (*F*) Transverse section of the central disk region showing ArSSP1-expressing cells in the coelomic epithelial lining of the peristomial membrane (arrowhead). (*G*) Higher-magnification image of the boxed region in *F*, showing stained cells in the coelomic epithelial lining of the peristomial membrane (arrowheads). (*H*) Transverse section of the central disk region showing stained cells in the esophagus (white arrowhead) and in the peristomial membrane (black arrowheads). (*I*) Transverse section of the central disk region showing stained cells in the cardiac stomach (arrowheads). (*J*) Higher-magnification image of the boxed region in *I*, showing round-shaped stained cells (arrowheads) adjacent to the visceral muscle layer. (*K*) Transverse section of the central disk region showing staining in the pyloric stomach. (*L*) Higher-magnification image of the boxed region in *K*, showing elongate-shaped stained cells in the mucosal layer (arrowhead). (*M*) Transverse section of the central disk region showing stained cells in the pyloric duct (black arrowheads) and in the coelomic epithelial lining of the apical muscle (white arrowheads). (*N*) Higher-magnification image of the boxed region in *M*, showing elongate-shaped stained cells in the mucosal layer (arrowhead). (*O*) Transverse section of an arm showing stained cells in a pyloric cecum. Elongate-shaped stained cells can be seen in the mucosal layer (arrowhead), and round-shaped stained cells (arrows) can be seen close to the position of the basiepithelial nerve plexus. (*P*) Longitudinal section of a tube foot showing stained cells at the junction between the stem and the disk region. (*Q*) Higher-magnification image of the boxed region in *P*, showing stained cells (arrowheads) are located around the basal nerve ring. (*R*) Transverse section of an ampulla of a tube foot showing stained cells in the coelomic epithelial lining. The *Inset* shows higher-magnification image of the boxed region, showing stained cells (arrowheads) in the coelomic epithelial lining of the ampulla. (*S*) Transverse section of the central disk region showing stained cells in the coelomic lining of the body wall (arrowheads) and the coelomic epithelial layer of the apical muscle (arrow). Abbreviations: AM, apical muscle; BNP, basiepithelial nerve plexus; BNR, basal nerve ring; CE, coelomic epithelium; CONR, circumoral nerve ring; CT, collagenous tissue; Di, disk; Ec, ectoneural region; Es; esophagus; Hy, hyponeural region; Lu, lumen; ML, muscle layer; MN, marginal nerve; Mu, mucosa; PD, pyloric duct; PM, peristomial membrane; RHS, radial hemal strand; RNC, radial nerve cord; St, stem. TF, tube foot; THS, transverse hemal strand; VML, visceral muscle layer. (Scale bars, 8 μm in *R*, *Inset*; 32 μm in *A*, *B*, *D*, *E*, *J*, and *L*; 60 μm in *A*, *Inset*, *C*, *G*, *H*, *K*, *N*, *O*, *Q*, *R*, and *S*; 120 μm in *F*, *I*, *M*, and *P*.)

Analysis of the distribution of ArSSP1 transcripts in *A. rubens* using messenger RNA (mRNA) in situ hybridization revealed expression in the nervous system, digestive system, tube feet, and body wall (Fig. 4). In the nervous system, ArSSP1-expressing cells were revealed in both the ectoneural and hyponeural regions of the radial nerve cords and circumoral nerve ring and in the marginal nerves (Fig. 4 *A–E*). ArSSP1-expressing cells were revealed in several regions of the digestive system, including the peristomial membrane (Fig. 4 *F–H*), esophagus (Fig. 4*H*), cardiac stomach (Fig. 4 *I* and *J*), pyloric stomach (Fig. 4 *K* and *L*), pyloric duct (Fig. 4 *M* and *N*), and pyloric caeca (Fig. 4*O*). In tube feet, ArSSP1-expressing cells were revealed at the junction between the stem and the disk regions proximal to the basal nerve ring (Fig. 4 *P* and *Q*) and in the coelomic lining of the ampulla (Fig. 4*R*). Lastly, ArSSP1-expressing cells were revealed in the coelomic lining of the body wall and apical muscle, a longitudinally orientated strand of muscle that runs along the midline of the inner surface of the aboral body wall in each arm (Fig. 4 *M* and *S*).

**Fig. 5.**
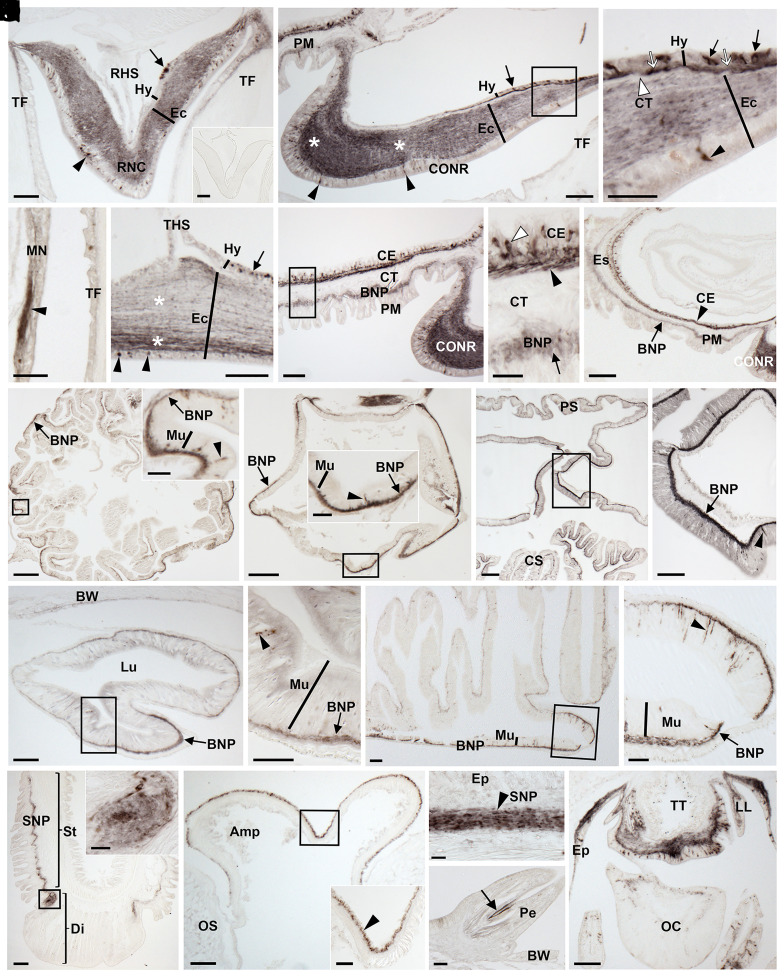
Localization of ArSS1 in *A. rubens* using immunohistochemistry. (*A*) Transverse section of a radial nerve cord showing ArSS1 immunoreactivity (immunostaining) in the hyponeural (arrow) and ectoneural (arrowhead) regions. The *Inset* shows that no staining is observed in sections of radial nerve cord incubated with affinity-purified ArSS1 antibodies preabsorbed with ArSS1 peptide, demonstrating the specificity of immunostaining observed in sections incubated with affinity-purified ArSS1 antibodies. (*B*) Transverse section of the circumoral nerve ring showing immunostained cells in the hyponeural region (arrow) and in the subcuticular epithelial layer of the ectoneural region (arrowheads); note the regional variation in the density of immunostained fibers in the ectoneural neuropile (asterisks). Immunostaining can also be seen here in the basiepithelial nerve plexus of the adjacent peristomial membrane. (*C*) Higher-magnification image of the boxed region in *B*, showing round-shaped monopolar immunostained cells (arrows) in the hyponeural region and immunostained fibers (white arrows) parallel to the collagenous tissue layer (white arrowhead), which is located between the hyponeural and ectoneural regions. (*D*) Immunostaining can be seen here in a marginal nerve; note the strongly immunostained fibers (arrowhead) emanating from the marginal nerve. (*E*) Parasagittal longitudinal section of a radial nerve cord showing immunostained cells in the hyponeural region (arrow) and in the subcuticular epithelial layer of the ectoneural region (arrowheads); note also the regional variation in the density of immunostained fibers in the ectoneural neuropile (asterisks). (*F*) Transverse section through the central disk region showing ArSS1 immunoreactivity (immunostaining) in the peristomial membrane and the adjacent circumoral nerve ring. (*G*) Higher-magnification image of the boxed region in *F*, showing immunostained cells in the coelomic epithelium (white arrowhead) and immunostained fibers in the underlying nerve plexus (black arrowhead). Immunostained fibers can also be seen here in the basiepithelial nerve plexus (arrow) underlying the external epithelial layer of the peristomial membrane. The collagenous tissue layer that separates the two nerve plexi is unstained. (*H*) Transverse section through the central disk region showing immunostaining in a longitudinal section of the esophagus and in the peristomial membrane and circumoral nerve ring. More specifically, immunostaining can be seen in the coelomic epithelium (arrowhead) and the basiepithelial nerve plexus (arrow). (*I*) Horizontal section of the cardiac stomach showing immunostained fibers in the basiepithelial nerve plexus (arrow) with regional variation in staining intensity. The *Inset* shows a higher-magnification image of the boxed region, showing immunostained bipolar shaped cells present in the mucosal layer (arrowhead) and immunostained fibers in the basiepithelial nerve plexus (arrow). (*J*) Horizontal section of the pyloric stomach showing immunostained fibers in the basiepithelial nerve plexus (arrow) with regional variation in staining intensity. The *Inset* shows a higher-magnification image of the boxed region, showing immunostained bipolar shaped cells in the mucosal layer (arrowhead) and immunostained fibers in the basiepithelial nerve plexus (arrow). (*K*) Transverse section through the central disk region showing intense immunostaining in the region of the digestive system linking the cardiac stomach with the pyloric stomach. (*L*) Higher-magnification image of the boxed region in *K*, showing immunostained bipolar-shaped cells present in the mucosal layer (arrowhead) and intense immunostaining in the basiepithelial nerve plexus (arrow). (*M*) Transverse section of a pyloric duct showing immunostaining in the basiepithelial nerve plexus on both the aboral and oral sides but with denser staining on the oral side (arrow). (*N*) Higher-magnification image of the boxed region in *M*, showing immunostained bipolar-shaped cells (arrowhead) present in the mucosal layer and immunostained fibers in the basiepithelial nerve plexus (arrow). (*O*) Transverse section of a starfish arm showing regional variation in the intensity of immunostaining in a pyloric cecum, with immunostaining most intense in the oral region that is continuous with the pyloric ducts. (*P*) Higher-magnification image of the boxed region in *O*, showing immunostained bipolar-shaped cells in the mucosal layer (arrowhead) and immunostaining in the basiepithelial nerve plexus (arrow). (*Q*) Longitudinal section of a tube foot showing ArSS1 immunoreactivity in the subepithelial nerve plexus along the length of the stem and extending into the basal nerve ring of the disk region. The *Inset* shows a higher-magnification image of the boxed region, showing immunostaining in the basal nerve ring of the disk region. (*R*) Parasagittal longitudinal section of an ampulla of a tube foot showing immunostaining in the coelomic epithelial lining of the ampulla. The *Inset* shows a higher-magnification image of the boxed region, showing immunostaining in the coelomic epithelial layer (arrowhead). (*S*) Transverse section of the aboral body wall showing immunostaining in the subepithelial nerve plexus beneath the external epithelial layer of aboral body wall. (*T*) Transverse section of the aboral body wall showing immunostaining (arrow) in a pedicellaria. (*U*) Transverse section of an arm tip, showing immunostaining in the terminal tentacle, lateral lappet, optic cushion, and the surrounding body wall epithelium. Abbreviations: Amp, ampulla; BNP, basi-epithelial nerve plexus; BW, body wall; CE, coelomic epithelium; CONR, circumoral nerve ring; CS, cardiac stomach; CT, collagenous tissue; Di, disk; Ec, ectoneural region; Ep, epithelium; Es, esophagus; Hy, hyponeural region; LL, lateral lappet; Lu, lumen; MN, marginal nerve; Mu, mucosa; OC, optic cushion; OS, ossicle; Pe, pedicellaria; PM, peristomial membrane; PS, pyloric stomach; RHS, radial hemal strand; RNC: radial nerve cord; SNP, subepithelial nerve plexus; St, stem; TF, tube foot. THS, transverse hemal strand; TT, terminal tentacle. (Scale bars, 12 μm in *Q*, *Inset*; 20 μm in *C*, *G*, *I*, *Inset*, and *R*, *Inset*; 32 μm in *D*, *J*, *Inset*, *N*, *P*, *S*, and *T*; 60 μm in *A*, *A*, *Inset*, *B*, *E*, *F*, *L*, *M*, *O*, *Q*, *R*, and *U*; 120 μm in *H*, *I*, and *K*; and 160 μm in *J*.)

To facilitate visualization of the expression of the mature ArSS1 peptide in *A. rubens*, a rabbit antiserum to this peptide was generated and characterized using an enzyme-linked immunosorbent assay (ELISA) (*SI Appendix*, Fig. S1 *A* and *B*). Then, ArSS1-specific antibodies were affinity purified from the antiserum (*SI Appendix*, Fig. S1*C*) and used for immunohistochemical analysis of ArSS1 expression in *A. rubens*. Consistent with the distribution of ArSSP1 transcripts in *A. rubens* (Fig. 4), ArSS1-immunoreactivity (ir) was detected in the nervous system, digestive system, tube feet, and body wall (Fig. 5). In the nervous system, ArSS1-ir was revealed in 1) cells located in the epithelial layer of the ectoneural region of the radial nerve cords and circumoral nerve ring and the underlying neuropile, with regional variation in the density of immunostained fibers (Fig. 5 *A*–*C* and *E*); 2) cells and their axonal processes located in the hyponeural region of the radial nerve cords and circumoral nerve ring (Fig. 5 *A–C* and *E*); and 3) the marginal nerve (Fig. 5*D*). In the digestive system, ArSS1-ir was revealed in the peristomial membrane (Fig. 5 *F–H*), esophagus (Fig. 5*H*), cardiac and pyloric regions of the stomach (Fig. 5 *I–L*), pyloric duct (Fig. 5 *M* and *N*), and pyloric caeca (Fig. 5 *O* and *P*). In tube feet, ArSS1-ir was revealed in the subepithelial nerve plexus of the podium (Fig. 5*Q*), the basal nerve ring of the disk (Fig. 5*Q*), and the coelomic epithelial layer of the ampulla (Fig. 5*R*). In the body wall, ArSS1-ir was revealed in basiepithelial nerve plexus beneath the external epithelium of the body wall (Fig. 5*S*) and in fibers that innervate muscles in the pedicellariae (Fig. 5*T*). Lastly, an extensive population of cells and fibers exhibiting ArSS1-ir were revealed in the arm tips localized in the terminal tentacle, lateral lappets, optic cushion, and the external epithelium of the body wall (Fig. 5*U*).

In summary, the overall expression pattern of ArSSP1/ArSS1 is not dissimilar to that of ArSSP2/ArSS2 and many other neuropeptides in *A. rubens*, as summarized in *SI Appendix*, Table S3. However, there are specific regional differences, as illustrated in *SI Appendix*, Fig. S2, which shows that ArSS1, but not ArSS2, is expressed by cells/fibers in the coelomic epithelial layer of tube foot ampullae.

### ArSS1 Causes Concentration-Dependent Contraction of Tube Foot, Apical Muscle, and Cardiac Stomach In Vitro Preparations from *A. rubens*.

**Fig. 6.**
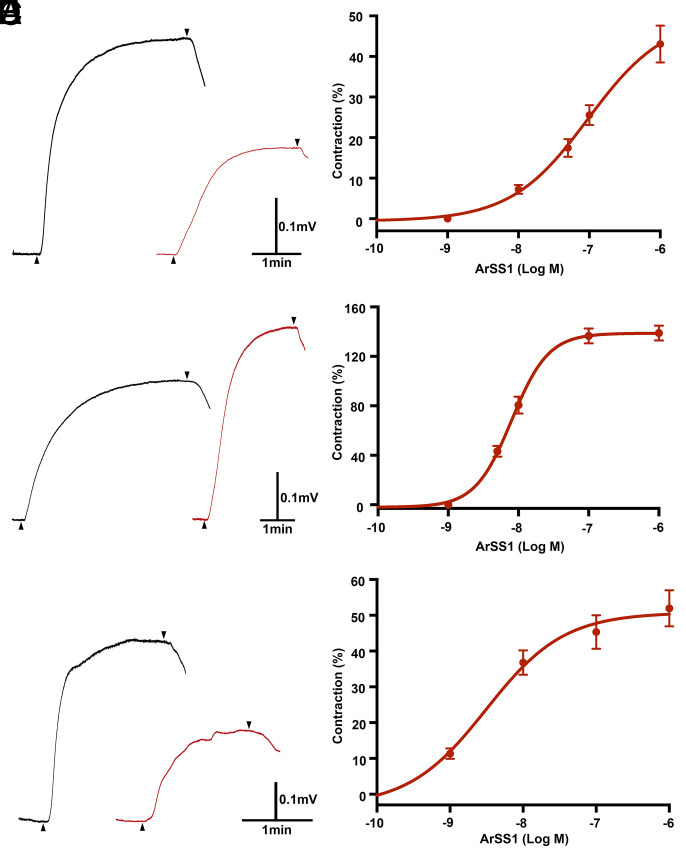
ArSS1 causes concentration-dependent contraction of tube foot, apical muscle, and cardiac stomach preparations from *A. rubens*. (*A*) Representative recordings showing that ACh (black) and ArSS1 (red) cause contraction of a tube foot preparation when tested at a concentration of 10^−6^ M. The upward-pointing arrowheads show when test agents were added, and the downward-pointing arrowheads show when the preparation was washed with seawater. (*B*) Graph showing that ArSS1 causes concentration-dependent contraction of tube foot preparations at concentrations ranging from 10^−8^ M to 10^−6^ M. The responses are expressed as the mean percentage (± SEM; *n* = 8) of the contraction induced by 10^−6^ M ACh. (*C*) Representative recordings showing that ACh (black) and ArSS1 (red) cause contraction of an apical muscle preparation when tested at a concentration of 10^−6^ M. The upward-pointing arrowheads show when test agents were added, and the downward-pointing arrowheads show when the preparation was washed with seawater. (*D*) Graph showing that ArSS1 causes concentration-dependent contraction of apical muscle preparations at concentrations ranging from 10^−8^ M to 10^−6^ M. The responses are expressed as the mean percentage (± SEM; *n* = 8) of the contraction induced by 10^−6^ M ACh. (*E*) Representative recordings showing that NGFFYamide (10^−7^ M; black) and ArSS1 (10^−6^ M; red) cause contraction of a cardiac stomach preparation. The upward-pointing arrowheads show when test agents were added, and the downward-pointing arrowheads show when the preparation was washed with seawater. (*F*) Graph showing that ArSS1 causes concentration-dependent contraction of cardiac stomach preparations at concentrations ranging from 10^−9^ M to 10^−6^ M. The responses are expressed as the mean percentage (± SEM; *n* = 8) of the contraction induced by 10^−7^ M NGFFYamide.

Informed by the expression of ArSSP1 (Fig. 4) and ArSS1 (Fig. 5) in the tube feet, apical muscle, and cardiac stomach of *A. rubens*, we investigated the effects of ArSS1 on in vitro preparations of these organs. To facilitate comparison with known myoactive substances in *A. rubens*, effects of ArSS1 were compared with the contracting effects of acetylcholine (ACh) on tube foot and apical muscle preparations (30) and the contracting effect of the neuropeptide NGFFYamide on cardiac stomach preparations (31). ArSS1 caused concentration-dependent contraction of tube foot preparations at concentrations between 10^−8^ M and 10^−6^ M. At the highest concentration tested (10^−6^ M), ArSS1 caused 43 ± 4.5% of ACh (10^−6^ M)–induced tube foot contraction (Fig. 6 *A* and *B*). Thus, ArSS1 was less effective than ACh as a contractant of tube foot preparations. ArSS1 caused concentration-dependent contraction of apical muscle preparations at concentrations between 5 × 10^−9^ M and 10^−6^ M. At 10^−7^ M, the contracting effect of ArSS1 on the apical muscle was 136 ± 6.0%, in comparison with the contracting effect of ACh at 10^−6^ M. Similarly, at 10^−6^ M, the contracting effect of ArSS1 on the apical muscle was 138 ± 6.0% in comparison with the contracting effect of ACh at 10^−6^ M (Fig. 6 *C* and *D*).Thus, at both 10^−7^ M and 10^−6^ M, ArSS1 was more effective than 10^−6^ M ACh in inducing contraction of apical muscle preparations. For cardiac stomach preparations, ArSS1 caused concentration-dependent contraction at concentrations between 10^−9^ M and 10^−6^ M. At the highest concentration tested (10^−6^ M), ArSS1 caused 52 ± 5.0% of the contraction induced by 10^−7^ M NGFFYamide (Fig. 6 *E* and *F*). Thus, ArSS1 was less effective than NGFFYamide as a cardiac stomach contractant.

Previous studies have revealed that in vivo injection of NGFFYamide in *A. rubens* triggers retraction of the everted cardiac stomach and inhibition of feeding behavior (31, 32). Because ArSS1, like NGFFYamide, causes contraction of the cardiac stomach in vitro, albeit with less efficacy, we investigated the effects of in vivo injection of ArSS1 on feeding-related behavior in *A. rubens*. Injection of ArSS1 (10 µL ⋅ 10^−3^ M) did not induce cardiac stomach retraction in *A. rubens*. Thus, the mean area of the everted cardiac stomach in water-injected animals (negative control) and ArSS1-injected animals was not significantly different over a period of 300 s from the time of injection (*SI Appendix*, Fig. S3). In contrast, in positive control experiments, NGFFYamide (10 µL ⋅ 10^−7^ M) triggered cardiac stomach retraction, with the area of the everted cardiac stomach 300 s after injection being 43 ± 2.5% of the area everted prior to injection (*SI Appendix*, Fig. S2), findings that are consistent with previously reported experiments with NGFFYamide (31). Experiments designed to investigate if ArSS1 affects feeding behavior in *A. rubens* revealed that, by comparison with control experiments (10 µL water), ArSS1 (10 µL ⋅ 10^−3^ M) did not cause a significant difference in the time elapsed before starfish touched or enclosed a mussel (*SI Appendix*, Fig. S4).

## Discussion

### Sequence Analysis of SS/ASTC-Type Neuropeptides and Precursors in Echinoderms Indicates That Chordate SS-Type Neuropeptides and Protostome ASTC-Type Neuropeptides Are Paralogous.

Chordate SS-type neuropeptides and protostome ASTC-type neuropeptides have the shared characteristics of being derived from the carboxyl-terminal region of precursor proteins and then being posttranslationally modified to acquire a disulphide bridge between two cysteine residues (12–16). Identification of two G protein–coupled receptors that mediate effects of ASTC in *Drosophila melanogaster* revealed that these proteins are closely related to vertebrate SS-type receptors (18). Accordingly, subsequent phylogenetic analysis of G protein–coupled neuropeptide receptors in the Bilateria has also revealed a close relationship between chordate SS-type receptors and protostome ASTC-type receptors (2, 3). Furthermore, it was recently proposed that protostome ASTC-type neuropeptides are orthologs of chordate SS-type neuropeptides (19).

Discovery of two SS/ASTC-type neuropeptide precursors (SSP1 and SSP2) in species belonging to the phylum Echinodermata provides a new perspective on the evolution of SS/ASTC-type neuropeptides (8, 20). Comparison of the sequences of echinoderm SS1-type and SS2-type neuropeptides with SS/ASTC-type neuropeptides from other taxa revealed that the SS1-type neuropeptides have an FXP motif (in which X is variable) in common with some protostome ASTC-type neuropeptides, while an FWK motif is a characteristic shared by some SS2-type neuropeptides and chordate-type SS-type neuropeptides (Fig. 1) (21). These findings suggest that echinoderm SS1-type neuropeptides are orthologs of protostome ASTC-type neuropeptides and that echinoderm SS2-type neuropeptides are orthologs of chordate SS-type neuropeptides. However, with sequence identity limited to a two- to three-residue motif, evolutionary convergence cannot be ruled out. Therefore, additional evidence of orthology has been sought. The exon–intron structure of genes encoding SS/ASTC-type neuropeptides has been compared in echinoderms and other bilaterians, but specific characteristics that distinguish SS-type neuropeptide genes and ASTC-type neuropeptide genes were not identified (21). Here, relationships between echinoderm SS/ASTC-type neuropeptides and SS/ASTC-type neuropeptides in other taxa were investigated using the CLANS method to compare the sequences of the protein precursors of these neuropeptides. Importantly, this revealed that vertebrate SS-type precursors and protostome ASTC-type precursors form two distinct clusters. Furthermore, echinoderm SS1-type precursors only have links with protostome ASTC-type precursors, and echinoderm SS2-type precursors only have links with chordate SS-type precursors (Fig. 2). Combined with the evidence provided by the sequence motif (FXP) that echinoderm SS1-type neuropeptides share with some protostome ASTC-type neuropeptides and the sequence motif (FWK) that some echinoderm SS2-type neuropeptides share with chordate SS-type neuropeptides (Fig. 1), these findings suggest that echinoderm SS1-type neuropeptides are orthologs of protostome ASTC-type neuropeptides and that echinoderm SS2-type neuropeptides are orthologs of chordate SS-type neuropeptides. Furthermore, our analysis of gene synteny at the chromosomal level revealed that chromosomes containing the ASTC/SS-type precursor gene in the mollusk *P. maximus* and the cephalochordate *B. floridae* have a much larger number of putative orthologs on the *A. rubens* chromosome containing the ArSSP2 gene (ArChr18) than on the *A. rubens* chromosome containing the ArSSP1 gene (ArChr6) (Fig. 3). These findings indicate that the *A. rubens* SS2 precursor gene, *B. floridae* SS precursor gene, and *P. maximus* ASTC precursor gene are located on chromosomes that are extensively derived from a common ancestral urbilaterian chromosome, which we infer would have contained the gene that is the common ancestor of bilaterian SS/ASTC-type precursor genes. Conversely, we infer that interchromosomal gene translocation may have resulted in the positioning of the ArSSP1 gene on a chromosome that has fewer orthologs on chromosomes containing the ASTC/SS-type precursor gene in the mollusk *P. maximus* and the cephalochordate *B. floridae.*

**Fig. 7.**
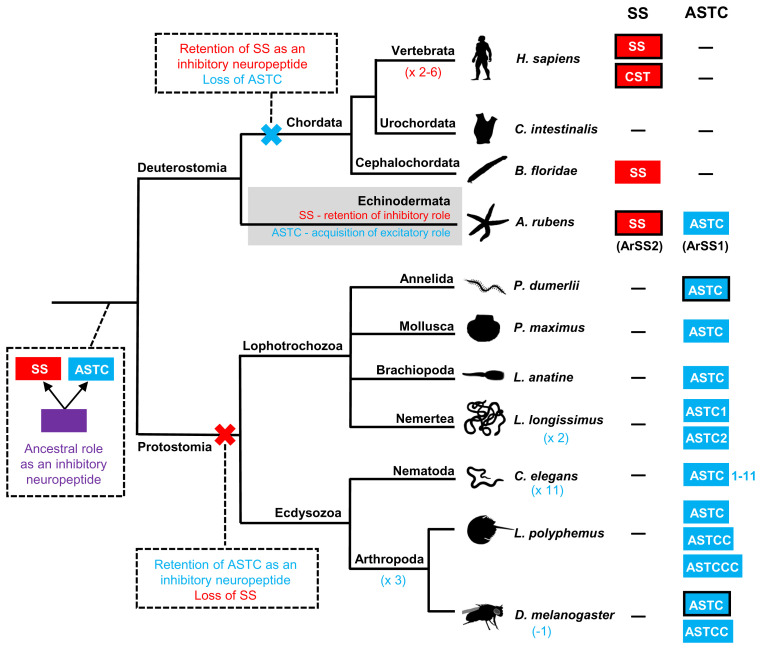
Diagram showing a model of the evolution of SS/ASTC-type neuropeptide signaling in the Bilateria, informed by the findings of this study. The occurrence of SS-type neuropeptides/precursors (red-filled boxes) and ASTC-type neuropeptide/precursors (blue-filled boxes) in different taxa is shown on the right side of the phylogenetic tree, with a dash (-) indicating absence of an SS-type and/or ASTC-type neuropeptide/precursor. Taxa in which the receptors for a SS-type or ASTC-type neuropeptide have been identified experimentally are identified by black outlining of boxes. Based on the phylogenetic distribution of SS/ASTC-type neuropeptides, it is inferred that duplication of a gene encoding an ancestral SS/ASTC-type neuropeptide (purple box) in a common ancestor of the Bilateria gave rise to genes encoding precursors of paralogous SS-type and ASTC-type neuropeptides, as shown at the root of the tree. ASTC-type and SS-type neuropeptides were lost in chordates and protostomes, respectively, because of functional redundancy as inhibitory neuropeptides. However, both ASTC-type (e.g., ArSS1) and SS-type (e.g., ArSS2) neuropeptides were retained in echinoderms because SS-type neuropeptides (e.g., ArSS2) were retained as inhibitory neuropeptides and ASTC-type neuropeptides (e.g., ArSS1) acquired a new role as excitatory neuropeptides. Lineage-specific genome/gene duplications (e.g., ×2) or gene losses (e.g., −1) that have given rise to variation in the number of SS/ASTC-type neuropeptides in different taxa are shown in parentheses. Species names are as follows: *H. sapiens (Homo sapiens), C. intestinalis (Ciona intestinalis), B. floridae (Branchiostoma floridae), A. rubens (Asterias rubens), P. dumerilii (Platynereis dumerilii), P. maximus (Pecten maximus), L. anatine (Lingula anatine)*, *L. longissimus (Lineus longissimus), C. elegans (Caenorhabditis elegans), L. polyphemus (Limulus polyphemus),* and *D. melanogaster (Drosophila melanogaster)*.

Collectively, our analysis of sequence data suggests that protostome ASTC-type neuropeptides are paralogs, not orthologs, of chordate SS-type neuropeptides. Importantly, this changes our perspective on the evolution of SS/ASTC-type neuropeptide signaling in the Bilateria because previously it has been proposed that protostome ASTC-type neuropeptides are orthologs of chordate SS-type neuropeptides (19). As illustrated in Fig. 7, the discovery of both SS-type and ASTC-type neuropeptide precursors in echinoderms suggests that duplication of a gene encoding an SS/ASTC-type neuropeptide precursor in a common ancestor of the Bilateria gave rise to paralogous genes encoding SS-type and ASTC-type neuropeptide precursors. Both of these neuropeptide precursor types were then retained in echinoderms (SSP2 and SSP1, respectively), but there was differential gene loss in protostomes and chordates, with chordates retaining SS-type precursors but losing ASTC-type precursors and with protostomes retaining ASTC-type precursors but losing SS-type precursors. Furthermore, the discovery that both SS-type and ASTC-type neuropeptides may occur in echinoderms has provided a unique opportunity to compare the physiological roles of these paralogous neuropeptides in an echinoderm.

### SS-Type and ASTC-Type Neuropeptides Have Opposing Myoinhibitory and Myoexcitatory Effects in an Echinoderm.

A detailed analysis of the expression of the SS-type neuropeptide ArSS2 in the starfish *A. rubens* was reported previously (21), which revealed a widespread pattern of expression in the central nervous system, digestive system, and body wall–associated tissues/organs. Furthermore, analysis of the pharmacological actions of ArSS2 revealed that it acts as a muscle relaxant, an action that is consistent with the actions of SS-type neuropeptides in vertebrates and ASTC-type neuropeptides in protostomes as inhibitory regulators of physiological processes (13, 14, 23–26). Here, the expression and actions of the ASTC-type neuropeptide ArSS1 in *A. rubens* were investigated. As with ArSS2, ArSS1 was found to have a widespread pattern of expression in the central nervous system, digestive system, and body wall–associated tissues/organs. In this respect, both ArSS1 and ArSS2 are similar to many other neuropeptides in *A. rubens* (30, 33–38) (see *SI Appendix*, Table S3 for a comparative overview of neuropeptide expression and action in *A. rubens*). However, it remains to be determined to what extent ArSS1, ArSS2, and other neuropeptides are coexpressed at the cellular level, which will require use of double-labeling methods. Nevertheless, specific regional differences in patterns of neuropeptide expression are observed (*SI Appendix*, Table S3). For example, expression of ArSS1, but not ArSS2, is revealed in cells/fibers in the coelomic epithelial layer of ampullae (*SI Appendix*, Fig. S2), fluid-filled contractile organs that control the protraction and retraction of the tube feet that enable locomotor activity in starfish. Accordingly, use of mRNA in situ hybridization revealed ArSSP1 transcripts in the coelomic epithelium of tube foot ampullae. Furthermore, the occurrence of neurons in coelomic epithelia is a known characteristic of echinoderm nervous systems (39), and cells with neurites and/or cells proximal to nerve plexi that are immunoreactive with antibodies to ArSS1 and to other neuropeptides are also present in the coelomic epithelial layer of other organs/tissues (e.g., peristomial membrane and apical muscle) in *A. rubens* (Fig. 5*G*) (30, 33–36, 40).

Informed by the expression of ArSSP1/ArSS1 in the cardiac stomach region of the digestive system, tube feet, and the body wall–associated apical muscle, the pharmacological effects of ArSS1 on in vitro preparations of these three neuromuscular tissues/organs were investigated. Interestingly, ArSS1 had a myoexcitatory effect on all three preparations, causing concentration-dependent contraction at physiologically relevant concentrations ranging from 10^−9^ M to 10^−6^ M. ArSS1 joins a growing list of neuropeptides that have been found to act as myoexcitatory neuropeptides in starfish, as summarized in *SI Appendix*, Table S3. However, the myoexcitatory effect of ArSS1 in *A. rubens* is interesting because hitherto it has been discovered that SS-type and ASTC-type neuropeptides in other taxa typically act as inhibitory regulators of physiological processes at the cellular level (13, 14, 23–26). There are reports of SS causing muscle contraction of guinea pig gastric smooth muscle cells, but investigation of the mechanisms of action indicate that this is a consequence of SS causing inhibition of the release of a muscle relaxant (e.g., vasoactive intestinal peptide) [i.e., SS is causing disinhibition (41, 42)]. The mechanisms by which ArSS1 causes muscle contraction in *A. rubens* are unknown. One possibility would be that it inhibits the release of a muscle relaxant, as has been reported for SS (41, 42). Alternatively, ArSS1 may have a direct excitatory effect on muscle in starfish. Further insights into this issue will be obtained if the receptor(s) that mediate the myoexcitatory effects of ArSS1 in *A. rubens* can be identified.

### The Myoexcitatory Effect of an ASTC-Type Neuropeptide in an Echinoderm Changes our Perspective on the Evolution of SS/ASTC-Type Neuropeptide Function.

Analysis of neural transcriptome sequence data from *A. rubens* enabled identification of three transcripts encoding proteins (ArSSR1-3) that are orthologs of SS/ASTC-type G protein–coupled receptors that have been characterized in other taxa. Furthermore, pharmacological characterization of ArSSR1-3 revealed that ArSS2 acts as a ligand for all three receptors, while ArSS1 does not act as a ligand for any of the receptors (21). Recognizing that neural transcriptome sequence data may provide an incomplete representation of SS/ASTC-type G protein–coupled receptors in *A. rubens*, we reported previously our analysis of the genome sequence of another starfish species, the crown-of-thorns starfish *A. planci* (43), which revealed that orthologs of ArSSR1-3 were the only SS/ASTC-type G protein–coupled receptors to be identified in this species (21). Subsequently, we have analyzed a chromosomal assembly of the genome sequence of *A. rubens* (National Center for Biotechnology Information [NCBI] accession no. PRJEB33974), and this has revealed that the ArSSR1-3 genes, which each are located on different chromosomes, are the only genes encoding SS/ASTC-type G protein–coupled receptors in this species. Therefore, we conclude that myoexcitatory effects of ArSS1 in *A. rubens* are mediated by as yet unknown signaling mechanisms for SS/ASTC-type neuropeptides. One possibility is that coexpression of an interacting protein alters the ligand-binding properties of one or more of the ArSSR1-3 proteins to preferentially bind ArSS1 instead of ArSS2. Alternatively, a hitherto uncharacterized family of G protein–coupled receptors may mediate effects of SS1-type peptides in echinoderms. Another possibility is that the myoexcitatory effect of ArSS1 in *A. rubens* is mediated by peptide-gated cation channels, which have been shown to mediate effects of some neuropeptides in other taxa (44–46).

How do the findings of this study impact our broader understanding of the evolution of SS/ASTC-type neuropeptide signaling? Our findings indicate that the SS-type and ASTC-type neuropeptide signaling systems are paralogous and evolved by duplication of an ancestral SS/ASTC-type signaling system in the common ancestor of the Bilateria (Fig. 7). It should be noted, however, that evidence for the existence of SS/ASTC-type signaling in a nonbilaterian phylum (Cnidaria) has been reported (47), but this is not supported by more comprehensive analyses of the phylogenetic distribution of neuropeptide receptors (2, 5). From a functional perspective, it is noteworthy that SS-type neuropeptides typically exert inhibitory effects in chordates and ASTC-type neuropeptides typically exert inhibitory effects in protostomes (13, 14, 23–26). This suggests that the hypothetical ancestral SS/ASTC-type signaling system in Urbilateria would likewise have had inhibitory roles. Therefore, the loss of ASTC-type neuropeptides and receptors in chordates and the loss of SS-type neuropeptides and receptors in protostomes may be consequences of functional redundancy of paralogous signaling systems. In this context, our discovery that both the SS-type and ASTC-type neuropeptides have been retained in echinoderms and have opposing roles as myoinhibitory and myoexcitatory agents is interesting. We speculate that both signaling systems may have been retained in echinoderms because ASTC-type neuropeptides acquired an excitatory role, and this was achieved by the evolution of a novel ligand-receptor signaling mechanism, which may be linked with interchromosomal translocation of the ASTC-type precursor gene in this lineage. Therefore, discovery of the receptor(s) that mediate the excitatory effects of ArSS1 in *A. rubens* represents a fascinating objective for future work. Furthermore, from an evolutionary perspective, it will also be of interest to determine the occurrence and characteristics of SS/ASTC-type neuropeptides in hemichordates and xenacoelomorphs, phyla recognized as being closely related to echinoderms within the proposed deuterostome clade Xenambulacraria (48). SS/ASTC-type receptors are present in species belonging to these phyla (e.g., GenBank accession no. XP_002731809) (5), but we and others (2, 3, 5) have yet to identify genes encoding precursors of SS/ASTC-type neuropeptides that are candidate ligands for these receptors. If this can be accomplished, it would reveal whether the occurrence of both ASTC-type and SS-type neuropeptides is a unique feature of echinoderms or a more extensive characteristic of the Xenambulacraria or Ambulacraria.

## Materials and Methods

### Animals.

Starfish (*A. rubens*) used for the anatomical and pharmacological experiments reported here were collected at low tide near Margate (Kent, United Kingdom) or were obtained from a fisherman based in Whitstable (Kent, United Kingdom) and then transported to an aquarium located in the School of Biological and Behavioural Sciences at Queen Mary University of London. The animals were maintained in circulating artificial seawater (Premium Reef-Salt, Tropical Marine Centre) at a salinity of 32‰ and at a temperature of ∼12 °C under a 12 h/12 h light/dark cycle. The starfish were fed on mussels (*Mytilus edulis*) that were also collected near Margate at low tide. Smaller juvenile specimens of *A. rubens* were also used for anatomical studies, and these were collected at the University of Gothenburg Sven Lovén Centre for Marine Infrastructure (Kristenberg, Sweden) and fixed in Bouin’s solution. Approval by an institutional review board was not required for this study because experimental research on starfish is not regulated.

### Sequence Alignment of Echinoderm SS-Type Peptides with SS/ASTC-Related Peptides in Other Taxa.

The amino acid sequences of ArSS1 and related peptides from other echinoderms were aligned with the sequences of ASTC-related neuropeptides from a variety of protostome species. Separately, the amino acid sequences of ArSS2 and related peptides from other echinoderms were aligned with the sequences of SS-type neuropeptides from chordates (see *SI Appendix*, Table S1 for a list of the sequences; identification of sequences was facilitated by reference to ref. 12 and BLAST analysis of sequence data on GenBank). This was accomplished using MAFFT 7 with the iterative refinement method set to L-INS-i and scoring matrix for amino acid sequences set to BLOSUM62, ensuring an optimal alignment. BOXSHADE software (https://github.com/mdbaron42/pyBoxshade) was used to highlight residues that are identical or are conservative substitutions in at least 60% of the aligned sequences.

### Investigation of the Relationships of the Echinoderm SS/ASTC-Type Neuropeptides with SS/ASTC-Type Peptides in Other Taxa Using CLANS to Compare the Sequences of Precursor Proteins.

Sequence alignment of ArSS1 and ArSS2 with other SS/ASTC-type peptides revealed that echinoderm SS1-type neuropeptides share more sequence similarities with protostome ASTC-type peptides and that SS2-type neuropeptides share more sequence similarities with chordate SS-type peptides. To enable further investigation of the relationships of SS1-type and SS2-type neuropeptides with SS/ASTC-type neuropeptides in other taxa, a database of neuropeptide precursors was produced, which included echinoderm SS1/SS2-type precursors, vertebrate SS/UII/MCH-type precursors, cephalochordate SS-type precursors, and protostome ASTC-type precursors (see *SI Appendix*, Table S2 for a list of the sequences; production of this database was facilitated by reference to ref. 12 and BLAST analysis of sequence data on GenBank). Then the CLANS method was employed for comparative analysis of the sequences of the neuropeptide precursors (22). An all-against-all BLAST was performed using the scoring matrix BLOSUM62, and linkage clustering was performed with an e-value of 1e-2 to identify coherent clusters. The clustering was first performed in three dimensions, and then the map was collapsed to two dimensions (2D) to enable generation of the diagram shown in Fig. 2.

### Comparative Analysis of the Gene Repertoire of Chromosomes Containing SS/ASTC-Type Genes in *A. rubens*, the Chordate *B. floridae*, and the Protostome *P. maximus*.

The sequences of the *A. rubens* chromosomes containing the ArSSP1 gene (chromosome 6; NCBI accession no. NC_047067.1) and the ArSSP2 gene (chromosome 18; NCBI accession no. NC_047079.1) were analyzed to investigate the occurrence of genes with putative orthologs located on the chromosomes that contain the ASTC-type precursor gene in the mollusk *P. maximus* (chromosome 16; NCBI accession no. NC_047030.1) and the SS-type precursor gene in the cephalochordate *B. floridae* (chromosome 14; NCBI accession no. NC_049992.1), respectively. This gene synteny analysis was carried out using the TBtools (Toolbox for biologists) version 1.098652 (49). BLAST and reciprocal BLAST, with the e-value set to 1e-20 and the number of hits restricted to one, were used to compare the sequences of proteins encoded by genes located on 1) *A. rubens* chromosome 6 and *P. maximus* chromosome 16 and 2) *A. rubens* chromosome 18 and *B. floridae* chromosome 14. Protein pairs identified as putative orthologs were curated to combine data for predicted proteins encoded by the same gene and to match results from reciprocal BLAST analysis. Pairs of genes identified as encoding putative orthologs are listed in Dataset S1, and to enable visualization, these were mapped on circular plots generated using Circos 0.69-6 (50)

### Localization of ArSSP1 Expression in *A. rubens* Using mRNA In Situ Hybridization.

A complementary DNA (cDNA) comprising the complete open reading frame of ArSSP1 from *A. rubens* was amplified and cloned into a pBluescript II SK+ vector, as reported previously (21). Here, the pBluescript II SK+ plasmid containing ArSSP1 cDNA was used as a template for synthesis of digoxigenin (DIG)-labeled antisense and sense probes for mRNA in situ hybridization. The methods employed for the generation of probes, fixation and sectioning of specimens of *A. rubens*, and visualization of ArSSP1 transcripts in sections of *A. rubens* were the same as reported for ArSSP2 previously (21). The concentration of antisense or sense probes used for mRNA in situ hybridization was 800 ng/mL.

### Production and Characterization of Antibodies to ArSS1.

ArSS1 (KCIGRFQPFSMPC with a disulphide bridge between the cysteines as determined by ref. 21) was custom synthesized by Peptide Protein Research Ltd. and used as an antigen to generate rabbit antisera to ArSS1. ArSS1 has an N-terminal lysine residue providing two reactive amine groups that were utilized for glutaraldehyde-mediated coupling to thyroglobulin (Sigma-Aldrich) as a carrier protein. Rabbit immunization and serum collection was performed by Charles River Biologics. To assess production of antibodies during the immunization protocol and following collection of a terminal bleed, ELISAs were carried out to test antisera for the presence of antibodies to ArSS1. The method employed for antiserum production and characterization was the same as described for generation of an antiserum to ArSS2 (21).

Antibodies to ArSS1 were affinity purified using AminoLink Plus Immobilization Kit (Thermo Fisher Scientific). A total of 1 mg ArSS1 was dissolved in 2 mL phosphate-buffered saline (PBS) and coupled to a column of beaded agarose. After washing with PBS twice, 2 mL ArSS1 antiserum was added to the column and incubated at room temperature for 2 h on a rocking table. The column was then washed six times with 5 mL PBS to remove any unbound proteins from the column. The bound antibodies were first eluted with 6 mL glycine buffer (5.4 mL 100 mM glycine [VWR] and 0.6 mL Tris [1 M, pH = 7.0]). Then the column was washed with 5 mL PBS six times, and bound antibodies were eluted with 6 mL triethylamine buffer (5.4 mL 100 mM triethylamine [Sigma-Aldrich] and 0.6 mL Tris [1 M, pH = 7.0]). The glycine eluate and triethylamine eluate were dialyzed separately in PBS at 4 °C for 48 h. After addition of 0.1% sodium azide (VWR), the dialyzed purified antibodies were stored at 4 °C. ELISA was used to assess the titer of antibodies to ArSS1 in the dialyzed eluates.

### Localization of ArSS1 in *A. rubens* Using Immunohistochemistry.

The methods employed for fixation and sectioning of specimens of *A. rubens* and visualization of ArSS1 expression in sections of *A. rubens* using immunohistochemistry were the same as reported for ArSS2 (21). To assess the specificity of immunostaining, preabsorption was performed by incubating 10 µL affinity-purified antibodies with 4 µL 1 mM ArSS1 that was then diluted in PBS to a total volume of 100 µL. Then, the mixture was placed on a rocking shaker at room temperature for 2 h and diluted to 1:10 in 5% normal goat serum in PBS containing 0.1% Tween-20 (NGS/PBST) for testing on starfish sections. Affinity-purified antibodies to ArSS1 were diluted to 1:10 in 5% goat serum/PBST and then used for immunohistochemistry.

### Imaging.

Photographs of sections processed for mRNA in situ hybridization or immunohistochemistry were captured using a QIClich CCD Color Camera (Qimaging) linked to a DMRA2 light microscope (Leica Microsystems) and Volocity version 6.3.1 image analysis software (Perkin-Elmer) running on an iMac computer (27 inch with operating system Yosemite, version 10.10).

### Analysis of the In Vitro Pharmacological Effects of ArSS1 on Tube Foot, Apical Muscle, and Cardiac Stomach Preparations from *A. rubens*.

Synthetic ArSS1 (KCIGRFQPFSMPC with a disulphide bridge between the cysteine residues) was custom synthesized (Peptide Protein Research Ltd.) to enable testing of its pharmacological effects on in vitro preparations of organs/muscles from *A. rubens*. The methods employed to dissect tube feet, apical muscles, and cardiac stomachs and then set them up as organ bath preparations were the same as reported for ArSS2 (21). Once set up in the organ bath, preparations were incubated in artificial seawater until a stable baseline state was achieved. ArSS1 was added into the organ bath to achieve final concentrations in the range of 10^−10^ M to 10^−6^ M, and preliminary tests revealed that ArSS1 caused contraction of all three preparations. To enable normalization of responses to ArSS1 in different preparations, a known contractant of tube foot and apical muscle preparations (ACh) was tested on each preparation at a concentration of 10^−6^ M (30). Likewise, to enable normalization of responses to ArSS1 in cardiac stomach preparations, a known cardiac stomach contractant (NGFFYamide) was tested on each preparation at a concentration of 10^−7^ M (31). Thus, in these experiments, the contracting effect of 10^−7^ M NGFFYamide or 10^−6^ M ACh was defined as 100%, and the contracting effect of ArSS1 was then calculated as a percentage of the effect of NGFFYamide or ACh. Concentration-response curves were generated using the nonlinear regression (curve fit) model in Prism 6 (GraphPad).

### Analysis of the In Vivo Pharmacological of Effects of ArSS1 on *A. rubens*.

When *A. rubens* feed, the cardiac stomach is relaxed and everted out of the mouth over the digestible soft tissues of prey (e.g., mussels) (51, 52). Previous studies have revealed that the neuropeptide NGFFYamide not only causes cardiac stomach contraction in vitro but also triggers cardiac stomach retraction in vivo (31) and regulates feeding behavior in *A. rubens* (32). Therefore, because ArSS1 also causes contraction of cardiac stomach preparations in vitro, we proceeded to investigate if ArSS1 also triggers cardiac stomach retraction and/or affects feeding behavior in *A. rubens*.

### Investigating if ArSS1 Triggers Cardiac Stomach Retraction in *A. rubens*.

Nine medium-sized starfish (8 to 12 cm in diameter) were starved for 1 wk to normalize their physiological status. To induce cardiac stomach eversion, starfish were placed in seawater containing 2% added MgCl_2_, which acts as a muscle relaxant in marine invertebrates (53) and typically causes cardiac stomach eversion in *A. rubens* within a period of about 30 min (31). A 50-µL syringe was used to inject test agents into the perivisceral coelom of starfish. For each starfish, the test agent was injected via two opposite sites (5 µL each) in the dorsal body wall of the arms proximal to the junctions with the central disk region. Care was taken to inject test agents into the perivisceral coelom and not into the cardiac stomach. Starfish were first injected with 10 µL distilled water (control) and video recorded using a camera (Canon EOS 700D) for 5 min. Then, the same starfish was injected with 10 µL 100 nmol NGFFYamide (positive control) or 10 µL 1 mM ArSS1 and video recorded for 5 min. ArSS1 and NGFFYamide were tested on six and three individuals, respectively. Static images from video recordings were captured at 30-s intervals from the time of injection, and the 2D area of the everted cardiac stomach was measured using ImageJ software (https://imagej.nih.gov/ij/). Retraction of the cardiac stomach was calculated as a percentage of the area of cardiac stomach everted at the time of injection, as described previously (31). Data were analyzed statistically using the mixed-effect regression model in Prism 6 (GraphPad).

### Investigating if ArSS1 Affects Feeding Behavior in *A. rubens*.

A total of 30 medium-sized starfish (8 to 12 cm in diameter) with a normal righting response (54) and showing normal feeding behavior on a mussel after 24 d of starvation were selected and kept in an aquarium for another 24 d starvation. Then, the starfish were transferred and kept individually in Plexiglass aquaria (27.5 × 19 × 19.6 cm) containing 6 L aerated seawater with the base covered by gravel (Tropical Marine Centre Gravel Coarse #5). The exterior of the sides and base of the aquarium tanks were covered with black plastic, as described previously (32). After 3 d acclimation, the control group comprising 14 individuals was injected with distilled water, while the test group comprising 16 individuals was injected with 10 µL ⋅ 10^−3^ M ArSS1. A total of 10 min after injection, one mussel (25 to 33 mm) without any encrusting organisms attached (55) was placed at one end of the tank, while the starfish was put at the opposite end with one arm touching the wall of the tank and the madreporite directed toward the mussel. The distance between the starfish and the mussel was about 15 cm. Then the starfish behavior was continuously observed for 3 h and checked every 15 min for another 2 h. A total of 24 h later, the tank was checked to determine whether or not the starfish had successfully fed on the mussel. Two starfish from the control group and one starfish from the test group that did not feed on a mussel were not used for data analysis. The time taken for starfish to first touch the mussel and then enclose the mussel was recorded and then compared for water-injected (control) and ArSS1-injected animals using Prism 6 (GraphPad) with statistical analysis (two-tailed Student’s *t* test).

## Supplementary Material

Supplementary File

Supplementary File

## Data Availability

*A. rubens* genome sequence data have been deposited in the NCBI BioProject database (PRJEB33974).
