# Supplementary material for: Somatostatin-type and allatostatin-C–type neuropeptides are paralogous and have opposing myoregulatory roles in an echinoderm
Source: Proc Natl Acad Sci U S A. 2022 Feb 10;119(7):e2113589119. doi: 10.1073/pnas.2113589119 (PMC8851493; doi:10.1073/pnas.2113589119)
Supplement: Supplementary File [file pnas.2113589119.sapp.pdf]

PNAS
www.pnas.org

Supplementary Information for

Somatostatin-type and allatostatin-C-type neuropeptides are paralogous and have opposing myoregulatory roles in an echinoderm

Ya Zhang[1], Luis Alfonso Yañez Guerra[1,2], Ana B. Tinoco[1], Nayeli Escudero Castelán[1], Michaela Egertová[1], Maurice R. Elphick[1*]

1. School of Biological and Behavioural Sciences, Queen Mary University of London, London E1 4NS, UK

2. Present address: Living Systems Institute, University of Exeter, Exeter EX4 4QD, UK

*Corresponding author: Maurice R. Elphick.

Email: m.r.elphick@qmul.ac.uk

This includes:

Figures S1-S4

Tables S1-S3

SI References

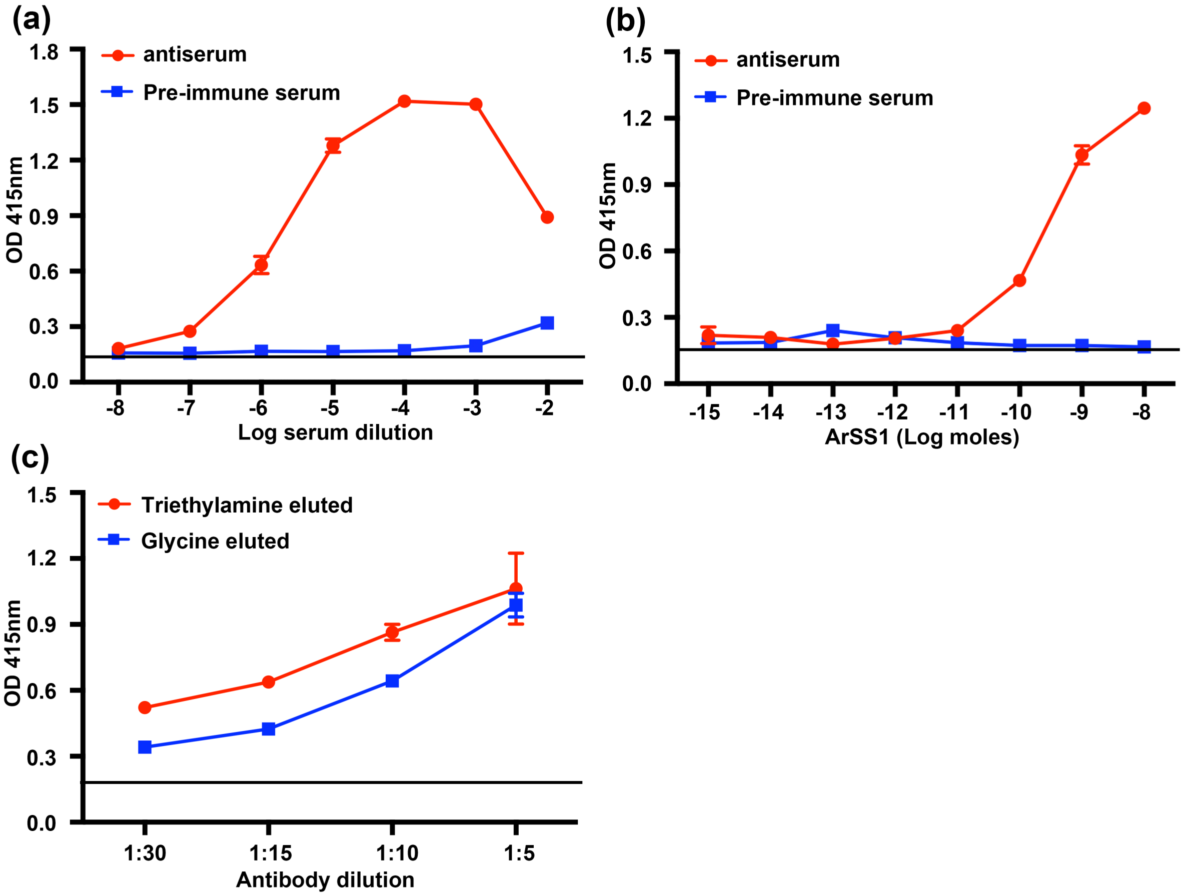

Fig. S1. Characterization of rabbit antiserum and affinity-purified rabbit antibodies to ArSS1 using an enzyme-linked immunosorbent assay (ELISA). (a) Incubation of antiserum (red) and pre-immune serum (blue) at dilutions between 10^{-8} and 10^{-2} with 0.1 nmol of ArSS1 reveals that absorbance values with the pre-immune serum are indistinguishable from the blank control without serum (black line), with the antiserum immunoreaction observed with dilutions from 10^{-7} to 10^{-2}. (b) Incubation of antiserum (red) and pre-immune serum (blue) at 10^{-4} dilution with between 10^{-15} and 10^{-8} moles of ArSS1 reveals that absorbance values with the pre-immune serum are indistinguishable from the blank control without serum (black line), with the antiserum immunoreaction observed with 10^{-10} to 10^{-8} moles of ArSS1. (c) Incubation of affinity-purified antibodies with 0.1 nmol of ArSS1 reveals that antibodies to ArSS1 can be detected in the trimethylamine eluate (red) and the glycine eluate (black) with dilutions from 1:30 to 1:5. The absorbance values with affinity-purified antibodies are distinguishably higher than the blank control (5 % goat serum/PBST; black line). All data points are mean values from two separate experiments performed in duplicate.

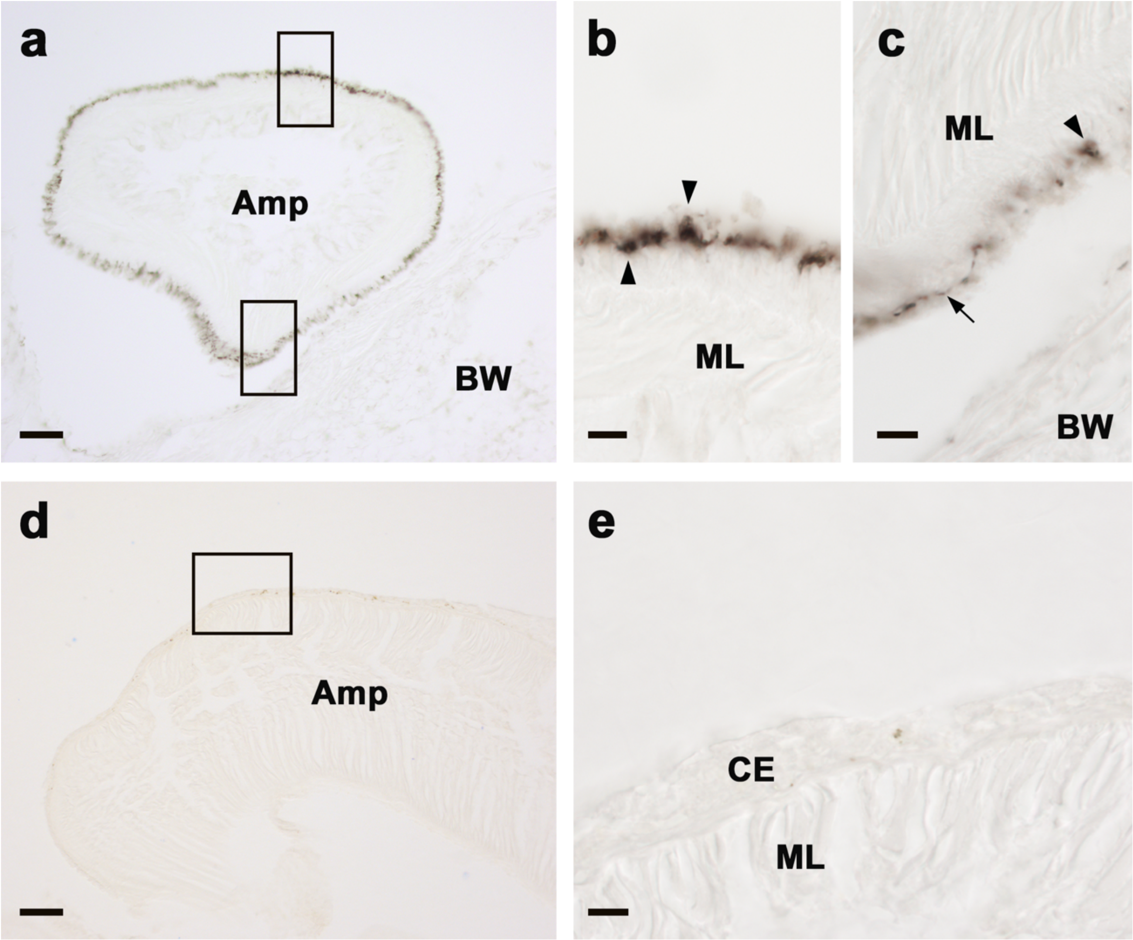

Fig. S2. **Comparative immunohistochemical analysis of ArSS1 and ArSS2 expression in tube foot ampullae of *A. rubens.*** (a) Transverse section of an arm showing ArSS1-immunoreactivity (immunostaining) in the coelomic epithelial layer of an ampulla. (b) Higher magnification image of the boxed upper region of (a), showing immunostained cells (arrowheads) in the coelomic epithelial layer. (c) Higher magnification image of the boxed lower region of (a), showing an immunostained cell (arrowhead) and varicose fibre (arrow) in the coelomic epithelial layer. (d) Transverse section of an arm showing absence of ArSS2 immunostaining in an ampulla. (e) Higher magnification image of the boxed region in (d), showing absence of ArSS2 immunostaining in the coelomic epithelial layer and the muscle layer of the ampulla. Abbreviations: Amp, ampulla; BW, body wall; CE, coelomic epithelium; ML, muscle layer. Scale bars: 6 μm in (b), (c), (e); 32 μm in (a), (d).

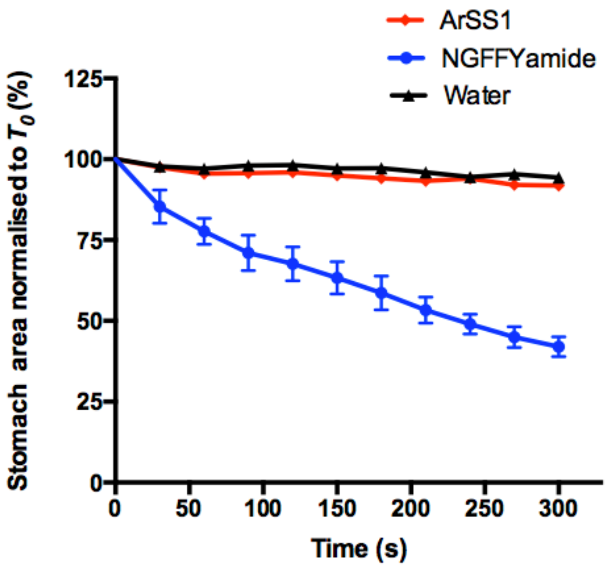

Fig. S3. ArSS1 does not induce cardiac stomach retraction in *A. rubens*. Retraction of the cardiac stomach was quantified by determining the area of the everted stomach as a percentage of the area of cardiac stomach everted at the time of injection (Time 0; T_0). The graph shows the mean area (± SEM) of the everted cardiac stomach expressed as a percentage of the area at T_0 and at 50 s intervals over a five-minute period following injection of distilled water (black; n = 6), 10 µl 1 mM ArSS1 (red; n = 6) or 10 µl 100 nmol NGFFYamide (blue; n = 3). Statistical analysis of these data by using mixed-effect regression model revealed no significance difference in slope between injection with ArSS1 and injection with water ($p = 0.0758$). In contrast NGFFYamide (used here as a positive control) clearly triggers cardiac stomach retraction, with significant difference in slope to injection with water or ArSS1 ($p<0.0001$).

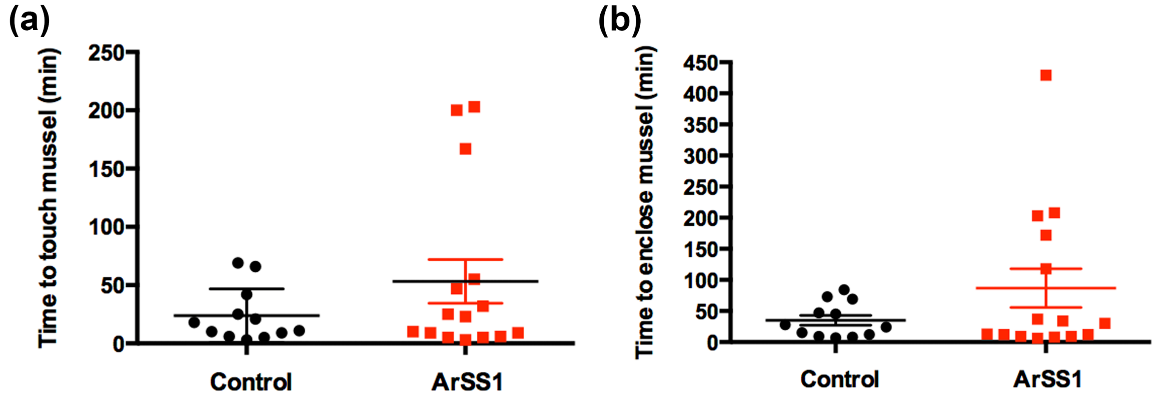

Fig. S4. (a) Injection of 10 µl of 10^{-3} M ArSS1 (red; n=15) did not cause a significant difference (t-test; p = 0.1905) in the time elapsed (minutes) before starfish touch a mussel in comparison with a control group injected with distilled water (black; n=12). (b) Injection of 10 µl of 10^{-3} M ArSS1 (red; n=15) did not cause a significant difference (t-test; p = 0.1605) in the time elapsed (minutes) before starfish enclose a mussel in comparison with a control group injected with distilled water (black; n=12). Data are shown as scatter plots and were analysed statistically using a two-tailed Student's t-test in Prism 6.

Table S1. Accession numbers of the precursor sequences used for the neuropeptide alignment in Figure 1.

**Species name**	**Neuropeptide precursor name**	**Accession number or reference**
Asterias rubens	Somatostatin1	ALJ99950.1
Acanthaster planci	Somatostatin1	XP_022111848.1
Ophionotus victoriae	Somatostatin1	XP_001176809.1
Strongylocentrotus purpuratus	Somatostatin1	(1)
Apostichopus japonicus	Somatostatin1	AWU78771.1
Drosophila melanogaster	Allatostatin C	NP_001162948.1
Limulus polyphemus	Allatostatin C	XP_022249713.1
Drosophila melanogaster	Allatostatin CC	NP_609483.2
Limulus polyphemus	Allatostatin CC	(2)
Limulus polyphemus	Allatostatin CCC	(2)
Caenorhabditis elegans	Allatostatin C1	(3)
Caenorhabditis elegans	Allatostatin C2	(3)
Caenorhabditis elegans	Allatostatin C3	(3)
Caenorhabditis elegans	Allatostatin C4	(3)
Caenorhabditis elegans	Allatostatin C5	(3)
Caenorhabditis elegans	Allatostatin C6	(4)
Caenorhabditis elegans	Allatostatin C7	(4)
Caenorhabditis elegans	Allatostatin C8	(5)
Caenorhabditis elegans	Allatostatin C9	(5)
Caenorhabditis elegans	Allatostatin C10	(5)
Caenorhabditis elegans	Allatostatin C11	(5)
Helobdella robusta	Allatostatin C	(3)
Platynereis dumerilii	Allatostatin C	AHB62362.1
Deroceras reticulatum	Allatostatin C	ARS01353
Charonia tritonis	Allatostatin C	AQS80487
Pecten Maximus	Allatostatin C	XP_033764281
Crassostrea gigas	Allatostatin C	XP_011412814
Lottia gigantea	Allatostatin C	(3)
Lingula anatine	Allatostatin C	XP_013393587.1
Lineus longissimus	Allatostatin C1	(6)
Lineus longissimus	Allatostatin C2	(6)
Asterias rubens	Somatostatin 2	MN257487
Acanthaster planci	Somatostatin 2	gbr.24.36.t1
Ophionotus victoriae	Somatostatin 2	(1)
Strongylocentrotus purpuratus	Somatostatin 2	(1)
Apostichopus japonicus	Somatostatin 2	(1)
Branchiostoma floridae	Somatostatin	(7)
Homo sapiens	Somatostatin	NP_001039.1
Mus musculus	Somatostatin	NM_009215.1
Gallus gallus	Somatostatin	CN210817.1
Danio rerio	Somatostatin	AAH76254.1
Oryzias latipes	Somatostatin	(8)
Takifugu rubripes	Somatostatin	(8)
Homo sapiens	Somatostatin (CST)	AF013252
Mus musculus	Somatostatin	AF050156.1
Gallus gallus	Somatostatin	(8)
Danio rerio	Somatostatin	(8)
Oryzias latipes	Somatostatin	(8)

Takifugu rubripes	Somatostatin	(8)
Danio rerio	Somatostatin	(8)
Oryzias latipes	Somatostatin	(8)
Takifugu rubripes	Somatostatin	(8)
Lepisosteus oculatus	Somatostatin	(8)
Lycopus americanus	Somatostatin	(8)
Danio rerio	Somatostatin	(8)
Oryzias latipes	Somatostatin	(8)
Takifugu rubripes	Somatostatin	(8)
Danio rerio	Somatostatin	(8)
Oryzias latipes	Somatostatin	(8)
Takifugu rubripes	Somatostatin	(8)
Danio rerio	Somatostatin	(8)
Lepisosteus oculatus	Somatostatin	(8)

Table S2. Accession numbers of the sequences used for CLANS analysis in Figure 2.

Species name	Gene name	Accession number or reference
Homo sapiens	SS	NP_001039.1
Homo sapiens	CST	NP_001293.3
Ficedula albicollis	SS	ENSFALT00000007767.1
Gallus gallus	SS	CN210817.1
Anolis carolinensis	SS	XP_003228868
Pelodiscus sinensis	SS	ENSPSIT00000016437.1
Pelophylax ridibundus	SS	P87384.1
Latimeria chalumnae	SS	XP 005992512.1
Protopterus annectens	SS	AAD39138.1
Epinephelus coioides	SS	AAU93565.1
Fundulus heteroclitus	SS	EV461374.1
Gadus morhua	SS	AAZ85702.1
Gasterosteus aculeatus	SS	KT235748
Haplochromis burtoni	SS	AAS97964.1
Lophius americanus	SS	P01169.1
Oncorhynchus mykiss	SS	CA364601.1
Oryzias latipes	SS	XP 004084505.1
Poecilia formosa	SS	ENSPFOT00000005658.1
Salmo salar	SS	CB509644.1
Takifugu rubripes	SS	ENSTRUG00000010048
Tetraodon nigroviridis	SS	KT235747
Astyanax mexicanus	SS	ENSAMXT00000006698.1
Carassius auratus	SS	Q9YGH5.1
Chitala chitala	SS	AAK97070.2
Clupea harengus	SS	XP 012673158.1
Danio rerio	SS	AAH76254.1
Gnathonemus petersii	SS	AF292652
Ictalurus punctatus	SS	CAA23877.1
Megalobrama pellegrini	SS	AAO92644.1
Pygocentrus nattereri	SS	XP 017539070.1
Acipenser transmontanus	SS	AAL13248.1
Acipenser sinensis	SS	FJ792687.1
Lepisosteus oculatus	SS	XP 006637937.1
Callorhinchus milii	SS	XP 007910586.1
Rhincodon typus	SS	XP 020365362.1

Scylorhinus canicula	SS	JX964750
Macaca fascicularis	SS	XP 015298230
Bos taurus	SS	NP 001191825
Mus musculus	SS	AAD51127.1
Sus scrofa	SS	NP 001191865
Anolis carolinensis	SS	XP 008119733.1
Latimeria chalumnae	SS	XP_006001361.1
Astyanax mexicanus	SS	ENSAMXT00000013558.1
Clupea harengus	SS	XP 012681486.1
Danio rerio	SS	ENSDART00000146411.2
Ictalurus punctatus	SS	XP 017341959.1
Pygocentrus nattereri	SS	XM 017706353.1
Lepisosteus oculatus	SS	NC023203.1
Callorhinchus milii	SS	XP 007900347.1
Rhincodon typus	SS	XP 020390988.1
Scyliorhinus canicula	SS	MK491629
Epinephelus coioides	SS	AAU93566.1
Gasterosteus aculeatus	SS	KT235756
Lycopus americanus	SS	P01170.2
Oncorhynchus mykiss	SS	Q91194.1
Oryzias latipes	SS	AU168379.1
Poecilotheria formosa	SS	ENSPFOT00000005672.2
Salmo salar	SS	DW531645.1
Salmo salar	SS	CB505007.1
Takifugu rubripes	SS	ENSTRUT00000025350.1
Tetraodon nigroviridis	SS	ENSTNIT00000001563.1
Xiphophorus maculatus	SS	XP_005808296.2
Ambystoma mexicanum	SS	ENSAMXT00000006711.1
Carassius auratus	SS	AAF15306.1
Catostomus commersonii	SS	AAK97071.2
Chitala chitala	SS	AAV48555.1
Clupea harengus	SS	XP 012693360.1
Ctenopharyngodon idella	SS	ACB69424.1
Danio rerio	SS	XP 694143.1
Ictalurus punctatus	SS	CK424149.1
Gnathonemus petersii	SS	AAV48556.1
Osteoglossum bicirrhosum	SS	AAK97067.1
Pantodon buchholzi	SS	AAK97068.2
Pygocentrus nattereri	SS	XP 017539071.1

Lepisosteus oculatus	SS	ENSLOCT00000011545.1
Callorhinchus milii	SS	XP 007910587.1
Rhincodon typus	SS	XP 020365363.1
Scyliorhinus canicula	SS	MK491630
Gasterosteus aculeatus	SS	KT235754
Oryzias latipes	SS	KT235752
Salmo salar	SS	CA039604.1
Takifugu rubripes	SS	NC018911.1
Tetraodon nigroviridis	SS	KT235753
Danio rerio	SS	NP 571802.1
Ictalurus punctatus	SS	P01172.1
Lepisosteus oculatus	SS	ENSLOCT00000011651.1
Latimeria chalumnae	SS	XP 005999859.1
Gasterosteus aculeatus	SS	ALD51533.1
Oncorhynchus nerka	SS	EV380196.1
Oreochromis niloticus	SS	XP 005464163.1
Oryzias latipes	SS	XP 020567335.1
Poecilotheria formosa	SS	XP 007551472.1
Takifugu rubripes	SS	XP 003968367.1
Tetraodon nigroviridis	SS	ALD51532.1
Xiphophorus maculatus	SS	XP 005804860.3
Ambystoma mexicanum	SS	XP 007243346.1
Danio rerio	SS	XP 001333082.1
Ictalurus punctatus	SS	XP 017327476.1
Pimephales promelas	SS	DT105503.1
Pygocentrus nattereri	SS	XP 017578562.1
Lepisosteus oculatus	SS	XP 006627411.2
Scyliorhinus canicula	SS	JX964751
Anas platyrhynchos	SS	XP_005021334.1
Taeniopygia guttata	SS	NP_001232404.1
Latimeria chalumnae	SS	XP 005994063.1
Protopterus annectens	SS	Q9W7E9.1
Epinephelus coioides	SS	AAU93567.1
Gadus Morhua	SS	XP_030211281.1
Gasterosteus aculeatus	SS	ENSGACG00000007077
Oncorhynchus mykiss	SS	CX153272.1
Osmerus mordax	SS	EL519905.1
Takifugu rubripes	SS	ENSTRUG00000012370
Tetraodon nigroviridis	SS	ENSTNIG00000015178

Astyanax mexicanus	SS	ENSAMXT00000013526.1
Carassius auratus	SS	AAD09631.1
Clupea harengus	SS	XP 012681579.1
Ctenopharyngodon idella	SS	ACB69425.1
Ictalurus punctatus	SS	XP_017343581.1
Pimephales promelas	SS	DT250237.1
Acipenser transmontanus	SS	AAL13249.1
Lepisosteus oculatus	SS	XP 006642047.1
Callorhinchus milii	SS	XP 007895974.1
Rhincodon typus	SS	XP 020368528.1
Scyliorhinus canicula	SS	JX964752
Branchiostoma floridae	SS	(7)
Asterias rubens	SS	MN257487
Acanthaster planci	SS	gbr.24.36.t1
Ophionotus victoriae	SS	(1)
Strongylocentrotus purpuratus	SS	(1)
Apostichopus japonicus	SS	(1)
Asterias rubens	SS	ALJ99950.1
Acanthaster planci	SS	XP_022111848.1
Ophionotus victoriae	SS	XP_001176809.1
Strongylocentrotus purpuratus	SS	(1)
Apostichopus japonicus	SS	AWU78771.1
Capitella teleta	ASTC	(3)
Helobdella robusta	ASTC	(3)
Lottia gigantea	ASTC	(3)
Crassostrea gigas	ASTC	(3)
Daphnia pulex	ASTC	(3)
Tribolium castaneum	ASTC	(3)
Caenorhabditis elegans	ASTC1	(3)
Caenorhabditis elegans	ASTC2	(3)
Caenorhabditis elegans	ASTC3	(3)
Caenorhabditis elegans	ASTC4	(3)
Caenorhabditis elegans	ASTC5	(3)
Caenorhabditis elegans	ASTC6	(4)
Caenorhabditis elegans	ASTC7	(4)
Caenorhabditis elegans	ASTC8	(5)
Caenorhabditis elegans	ASTC9	(5)
Caenorhabditis elegans	ASTC10	(5)
Caenorhabditis elegans	ASTC11	(5)

Drosophila melanogaster	ASTCC	NP_609483.2
Drosophila melanogaster	ASTC	NP 001162948.1
Strigamia maritima	ASTC	(2)
Strigamia maritima	ASTCC	(2)
Strigamia maritima	ASTCCC	(2)
Frankliniella occidentalis	ASTC	KAE8745392.1
Nasonia vitripennis	ASTC	ADM26612.1
Odontomachus brunneus	ASTC	XP_032688105.1
Cydia pomonella	ASTC	QDK59880.1
Formica exsecta	ASTC	XP_029677371.1
Acyrthosiphon pisum	ASTC	XP_003244026.1
Nylanderia fulva	ASTC	XP_029159605.1
Ooceraea biroi	ASTC	XP_011338324.1
Mizuhopecten yessoensis	ASTC	AXN93471.1
Camponotus floridanus	ASTC	XP_011252628.1
Harpegnathos saltator	ASTC	XP_011138699.1
Plautia stali	ASTC	BAV78790.1
Aplysia californica	ASTC	ADX20596.1
Neocaridina denticulata	ASTC	AIY69122.1
Nilaparvata lugens	ASTC	BAO00971.1
Helicoverpa armigera	ASTC	AGH25547.1
Tribolium castaneum	ASTC	EFA09152.2
Bombyx mori	ASTC	NP_001124356.1
Clostera anastomosis	ASTC	AEM44669.1
Scylla paramamosain	ASTC	QDQ19208.1
Charonia tritonis	ASTC	AQS80487
Deroceras reticulatum	ASTC	ARS01353
Aplysia californica	ASTC	XP_005112794_1
Crassostrea gigas	ASTC	XP_011412814
Crassostrea virginica	ASTC	XP_022344895_1
Pecten maximus	ASTC	XP_033764281
Mizuhopecten yessoensis	ASTC	XP_021356393_1
Neocaridina denticulata	ASTC	AIY69122_1
Penaeus vannamei	ASTC	XP_027213118_1
Pandalus japonicus	ASTC	AFV91540_1
Scylla paramamosain	ASTC	QDQ19208_1
Hyalella azteca	ASTC	XP_018013863_1
Trinorchestia longiramus	ASTC	KAF2351638_1
Trinorchestia longiramus	ASTC	KAF2358496_1

Ctenocephalides felis	ASTC	XP_026481520_1
Clunio marinus	ASTC	CRK94328_1
Amphibalanus amphitrite	ASTC	KAF0305242_1
Limulus polyphemus	ASTC	XP_022249713_1
Lychas mucronatus	ASTC	ABY26679_1
Thrips palmi	ASTC	XP_034242426_1
Propsilocerus akamusi	ASTC	QGW50614_1
Drosophila albomicans	ASTC	XP_034098787_1
Stomoxys calcitrans	ASTC	XP_013118248_1
Frankliniella occidentalis	ASTC	XP_026282364_1
Drosophila kikkawai	ASTC	XP_017032718_1
Drosophila kikkawai	ASTC	XP_017032715_1
Varroa jacobsoni	ASTC	XP_022691927_1
Drosophila obscura	ASTC	XP_022214727_1
Drosophila grimshawi	ASTC	EDW03810_1
Drosophila takahashii	ASTC	XP_017014908_1
Aedes albopictus	ASTC	XP_029729533_1
Drosophila ananassae	ASTC	XP_014761510_1
Drosophila elegans	ASTC	XP_017110281_1
Drosophila eugracilis	ASTC	XP_017076362_1
Drosophila ficusphila	ASTC	XP_017059357_1
Drosophila innubila	ASTC	XP_034474889_1
Scaptodrosophila lebanonensis	ASTC	XP_030384485_1
Musca domestica	ASTC	XP_005184918_1
Anopheles gambiae	ASTC	XP_001689183_1
Anopheles darlingi	ASTC	ETN61475_1
Photinus pyralis	ASTC	KAB0794209_1
Sarcophaga bullata	ASTC	TMW48585_1
Drosophila arizonae	ASTC	XP_017858602_1
Drosophila busckii	ASTC	XP_017848162_1
Tribolium castaneum	ASTC	NP_001137205_1
Galendromus occidentalis	ASTC	XP_018495151_1
Limulus polyphemus	ASTCC	(2)
Anopheles sinensis	ASTC	KFB49259_1
Drosophila mauritiana	ASTC	XP_033161907_1
Drosophila willistoni	ASTC	XP_002067656_1
Zeugodacus cucurbitae	ASTC	XP_011178321_1
Bactrocera oleae	ASTC	XP_014086382_1
Bactrocera latifrons	ASTC	XP_018787536_1

Limulus polyphemus	ASTCCC	(2)
Stegodyphus dumicola	ASTC	XP_035230852_1
Samia ricini	ASTC	AAY68369_1
Stegodyphus mimosarum	ASTC	KFM78946_1
Drosophila virilis	ASTC	XP_002051772_1
Bactrocera oleae	ASTC	XP_014086384_1
Vanessa tameamea	ASTC	XP_026492179_1
Ceratitis capitata	ASTC	XP_020714951_1
Amyelois transitella	ASTC	XP_013186846_1
Clostera anastomosis	ASTCC	(9)
Apis mellifera	ASTCC	(10)
Apis mellifera	ASTCCC	(2)
Arctia plantaginis	ASTC	CAB3261401_1
Ostrinia furnacalis	ASTC	XP_028171302_1
Plutella xylostella	ASTC	NP_001295989_1
Chilo suppressalis	ASTC	ALM30301_1
Spodoptera frugiperda	ASTC	Q868F8_1
Hyposmocoma kahamanoa	ASTC	XP_026327560_1
Aphantopus hyperantus	ASTC	XP_034831767_1
Heliothis virescens	ASTC	PCG77404_1
Bombyx mandarina	ASTC	XP_028043051_1
Bicyclus anynana	ASTC	XP_023936107_1
Galleria mellonella	ASTC	XP_026761889_1
Operophtera brumata	ASTC	KOB78759_1
Papilio xuthus	ASTC	XP_013164626_1
Danaus plexippus	ASTC	XP_032517390_1
Mythimna unipuncta	ASTC	AAA93257_1
Papilio machaon	ASTC	XP_014367295_1
Agrotis ipsilon	ASTC	C0HKS0_1
Manduca sexta	ASTC	XP_030040870_1
Cydia pomonella	ASTC	QDK59880_1
Rhagoletis zephyria	ASTC	XP_017473348_1
Papilio polytes	ASTC	XP_013145137_1
Spodoptera exigua	ASTC	AXY04234_1
Lucilia cuprina	ASTC	XP_023293632_1
Asbolus verrucosus	ASTC	RZC32467_1
Nicrophorus vespilloides	ASTC	XP_017776616_1
Parasteatoda tepidariorum	ASTC	XP_015908718_1
Parasteatoda tepidariorum	ASTC	XP_015908719_1

Parasteatoda tepidariorum	ASTC	XP_015908717_1
Pieris rapae	ASTC	XP_022129619_1
Anoplophora glabripennis	ASTC	XP_018572075_1
Trichoplusia ni	ASTC	XP_026734642_1
Daphnia magna	ASTC	KZS12162_1
Contarinia nasturtii	ASTC	XP_031623836_1
Rhynchophorus ferrugineus	ASTC	QGA72540_1
Culex pipiens	ASTC	AHG94988_1
Aethina tumida	ASTC	XP_019881141_1
Dendroctonus ponderosae	ASTC	XP_019760338_1
Lingula anatina	ASTC	XP_013393587.1
Novocrania anomala	ASTC	(6)
Cephalothrix hongkongiensis	ASTC	(6)
Tubulanus polymorphus	ASTC	(6)
Lineus Longissimus	ASTC1	(6)
Cerebratulus spec	ASTC1	(6)
Lineus lacteus	ASTC1	(6)
Lineus ruber	ASTC1	(6)
Riseriellus occultus	ASTC	(6)
Cerebratulus marginatus	ASTC	(6)
Malacobdella grossa	ASTC	(6)
Notospermus geniculatus	ASTC	(6)
Cerebratulus spec	ASTC2	(6)
Lineus lacteus	ASTC2	(6)
Lineus longissimus	ASTC2	(6)
Lineus ruber	ASTC2	(6)
Nipponemertes spec	ASTC	(6)
Homo sapiens	UII	NP_006777.1
Homo sapiens	UII	NP_068835.1
Danio rerio	UII	NP_998013.2
Rattus norvegicus	UII	NP_062033.1
Pelophylax ridibundus	UII	P33715.2
Rattus norvegicus	UII	NP_596876.2
Sus scrofa	UII	NP_999308.1
Macaca mulatta	UII	NP_001028067.1
Phaethon lepturus	UII	XP_010282914.1
Macaca mulatta	UII	XP_028699396.1
Vulpes vulpes	UII	XP_025862965.1
Callorhinus ursinus	UII	XP_025724981.1

Takifugu rubripes	UII	NP_001072085.1
Cariama cristata	UII	XP_009698098.1
Xenopus laevis	UII	NP_001267509.1
Ovis aries	UII	XP_027821791.1
Gasterosteus aculeatus	UII	ALD51541.1
Tetraodon nigroviridis	UII	ALD51540.1
Aptenodytes forsteri	UII	KFM09588.1
Corvus brachyrhynchos	UII	KFO54906.1
Homo_sapiens	URP	BAC98929.1
Rattus_norvegicus	URP	NP_937766.1
Mus_musculus	URP	NP_937809.1
Danio_rerio	URP	NP_001245247.1
Danio_rerio	URP	NP_001076411.1
Hippoglossus stenolepis	URP	XP_035006504.1
Epinephelus lanceolatus	URP	XP_033471682.1
Anguilla_japonica	URP	BAJ51782.1
Homo sapiens	MCH	AAA63214.1
Mus_musculus	MCH	NP_084247.1
Oreochromis_mossambicus	MCH	P49794.1
Oncorhynchus keta	MCH	P19713.1

Neuro-peptide	Nervous system					Digestive system					Locomotory systems		
	RNC Ec	RNC Hy	CONR Ec	CONR Hy	PM	Es	CS	PS	PD	PC	TF Pod	TF Amp	AM
**ArSS1**	✓	✓	✓	✓	✓	✓	✓	✓	✓	✓	✓	✓	✓
**ArSS2**	✓	✓	✓	✓	✓		✓	✓	✓	✓	✓		
NGFFYa (*)	✓		✓				✓	✓	✓		✓		✓
ArSK/CCK1,2	✓	✓	✓	✓	✓	✓	✓	✓	✓	✓	✓		✓
ArGnRH	✓	✓	✓	✓	✓	✓	✓	✓	✓	✓	✓		✓
ArCRZ (*)	✓		✓				✓	✓	✓		✓		✓
Asterotocin	✓		✓		✓	✓	✓	✓	✓		✓		✓
SALMFa1 (+)	✓	✓	✓	✓		✓	✓	✓		✓	✓		✓
SALMFa2 (+)	✓		✓			✓	✓	✓		✓	✓		
ArPPLN1b/ SMP	✓	✓	✓	✓	✓	✓	✓	✓	✓	✓	✓	✓	✓
ArPPLN2h	✓	✓	✓	✓	✓	✓	✓	✓	✓	✓	✓	✓	
ArLQ (*)	✓		✓				✓	✓			✓		
ArCT	✓	✓	✓	✓	✓	✓	✓	✓	✓	✓	✓	✓	✓
ArCRH	✓	✓	✓	✓	✓	✓	✓	✓	✓	✓	✓	✓	✓

KEY:

✓ = expression reported

In vitro pharmacological effects reported = Myoexcitatory effect Myoinhibitory effect No effect

Table S3 Comparison of the expression patterns and pharmacological actions of ArSS1, ArSS2 and other neuropeptides that have been characterised in *Asterias rubens*. Reports of expression are based on use of mRNA *in situ* hybridisation and immunohistochemical methods, with the exception of neuropeptides labelled with an asterisk (*) where only mRNA *in situ* hybridisation has been employed and neuropeptides labelled with a cross (+) where only immunohistochemistry has been employed. Anatomical abbreviations: AM, apical muscle; CONR Ec; circumoral nerve ring ectoneural region; CONR Hy, circumoral nerve ring hyponeural region; CS, cardiac stomach; Es, esophagus; PC, pyloric caeca; PD, pyloric duct; PM, peristomial membrane; PS, pyloric stomach; RNC Ec; radial nerve cord ectoneural region; RNC Hy radial nerve cord hyponeural region; TF Amp, tube foot ampulla; TF Pod, tube foot podium.The findings summarised in this table are derived from the following publications, which are listed below: ArSS1 (this paper); ArSS2 (11); NGFFYamide (12); ArSK/CCK1,2 (13); ArGnRH (14); ArCRZ (14); Asterotocin (15); SALMFamide-1 (16-18) ; SALMFamide-2 (17-20); ArPPLN1b/SMP (21); ArPPLN2h (22); ArLQ (23); ArCT (24); ArCRH (25, 26).

SI References

1. M. Zandawala *et al.*, Discovery of novel representatives of bilaterian neuropeptide families and reconstruction of neuropeptide precursor evolution in ophiuroid echinoderms. *Open Biol* **7** (2017).
2. J. A. Veenstra, Allatostatins C, double C and triple C, the result of a local gene triplication in an ancestral arthropod. *Gen Comp Endocrinol* **230-231**, 153-157 (2016).
3. O. Mirabeau, J. S. Joly, Molecular evolution of peptidergic signaling systems in bilaterians. *Proc Natl Acad Sci U S A* **110**, E2028-2037 (2013).
4. S. Van Bael *et al.*, A Caenorhabditis elegans Mass Spectrometric Resource for Neuropeptidomics. *J Am Soc Mass Spectrom* **29**, 879-889 (2018).
5. F. M. McKay *et al.*, In silico analyses of neuropeptide-like protein (NLP) profiles in parasitic nematodes. *Int J Parasitol* 10.1016/j.ijpara.2021.07.002 (2021).
6. D. Thiel, L. A. Y. Guerra, M. Franz-Wachtel, A. Hejnol, G. Jekely, Nemertean, brachiopod and phoronid neuropeptidomics reveals ancestral spiralian signalling systems. *Mol Biol Evol* 10.1093/molbev/msab211 (2021).
7. D. C. Semmens *et al.*, Transcriptomic identification of starfish neuropeptide precursors yields new insights into neuropeptide evolution. *Open Biol* **6**, 150224 (2016).
8. H. Tostivint *et al.*, Molecular evolution of GPCRs: Somatostatin/urotensin II receptors. *J Mol Endocrinol* **52**, T61-86 (2014).
9. Y. Q. Dong, Z. Y. Wang, T. Z. Jing, Functional characterization of ASTC (allatostatin C) and ASTCC (allatostatin double C) in Clostera anastomosis (Lepidoptera: Notodontidae). *Gene* **598**, 1-8 (2017).
10. J. A. Veenstra, Allatostatin C and its paralog allatostatin double C: the arthropod somatostatins. *Insect Biochem Mol Biol* **39**, 161-170 (2009).
11. Y. Zhang *et al.*, Molecular and functional characterization of somatostatin-type signalling in a deuterostome invertebrate. *Open Biol* **10**, 200172 (2020).
12. A. B. Tinoco *et al.*, Characterization of NGFFYamide Signaling in Starfish Reveals Roles in Regulation of Feeding Behavior and Locomotory Systems. *Front Endocrinol (Lausanne)* **9**, 507 (2018).
13. A. B. Tinoco *et al.*, Ancient role of sulfakinin/cholecystokinin-type signalling in inhibitory regulation of feeding processes revealed in an echinoderm. *Elife* **10** (2021).
14. S. Tian, M. Egertova, M. R. Elphick, Functional Characterization of Paralogous Gonadotropin-Releasing Hormone-Type and Corazonin-Type Neuropeptides in an Echinoderm. *Front Endocrinol (Lausanne)* **8**, 259 (2017).
15. E. A. Odekunle *et al.*, Ancient role of vasopressin/oxytocin-type neuropeptides as regulators of feeding revealed in an echinoderm. *BMC Biol* **17**, 60 (2019).
16. S. J. Moore, M. C. Thorndyke, Immunocytochemical mapping of the novel echinoderm neuropeptide SALMFamide 1 (S1) in the starfish Asterias rubens. *Cell Tissue Res* **274**, 605-618 (1993).
17. R. Melarange, M. R. Elphick, Comparative analysis of nitric oxide and SALMFamide neuropeptides as general muscle relaxants in starfish. *J Exp Biol* **206**, 893-899 (2003).
18. R. Melarange, D. J. Potton, M. C. Thorndyke, M. R. Elphick, SALMFamide neuropeptides cause relaxation and eversion of the cardiac stomach in starfish. *Proc Biol Sci* **266**, 1785-1789 (1999).
19. S. J. Newman, M. R. Elphick, M. C. Thorndyke, Tissue distribution of the SALMFamide neuropeptides S1 and S2 in the starfish Asterias rubens using novel monoclonal and polyclonal antibodies. I. Nervous and locomotory systems. *Proc Biol Sci* **261**, 139-145 (1995).
20. S. J. Newman, M. R. Elphick, M. C. Thorndyke, Tissue distribution of the SALMFamide neuropeptides S1 and S2 in the starfish Asterias rubens using novel monoclonal and polyclonal antibodies. II. Digestive system. *Proc Biol Sci* **261**, 187-192 (1995).

21. M. Lin, M. Egertova, C. G. Zampronio, A. M. Jones, M. R. Elphick, Pedal peptide/orcokinin-type neuropeptide signaling in a deuterostome: The anatomy and pharmacology of starfish myorelaxant peptide in Asterias rubens. *J Comp Neurol* **525**, 3890-3917 (2017).
22. M. Lin, M. Egertova, C. G. Zampronio, A. M. Jones, M. R. Elphick, Functional characterization of a second pedal peptide/orcokinin-type neuropeptide signaling system in the starfish Asterias rubens. *J Comp Neurol* **526**, 858-876 (2018).
23. L. A. Yanez-Guerra *et al.*, Discovery and functional characterisation of a luqin-type neuropeptide signalling system in a deuterostome. *Sci Rep* **8**, 7220 (2018).
24. W. Cai *et al.*, Biochemical, Anatomical, and Pharmacological Characterization of Calcitonin-Type Neuropeptides in Starfish: Discovery of an Ancient Role as Muscle Relaxants. *Front Neurosci* **12**, 382 (2018).
25. W. Cai (2018) Discovery and functional characterisation of calcitonin-type and CRH-type neuropeptide signalling systems in an echinoderm. PhD thesis. in *School of Biological & Chemical Sciences* (Queen Mary University of London, London, UK), p 316.
26. W. Cai, M. Egertova, C. G. Zampronio, A. M. Jones, M. R. Elphick, Molecular identification and cellular localisation of a corticotropin-releasing hormone type neuropeptide in an echinoderm. *Neuroendocrinology* 10.1159/000517087 (2021).
